# Acknowledgement to Reviewers of *Sensors* in 2016

**DOI:** 10.3390/s17010128

**Published:** 2017-01-10

**Authors:** Sensors Editorial Office

**Affiliations:** MDPI AG, St. Alban-Anlage 66, 4052 Basel, Switzerland; sensors@mdpi.com

The editors of *Sensors* would like to express their sincere gratitude to the following reviewers for assessing manuscripts in 2016.

We greatly appreciate the contribution of expert reviewers, which is crucial to the journal’s editorial process. We aim to recognize reviewer contributions through several mechanisms, of which the annual publication of reviewer names is one. Reviewers receive a voucher entitling them to a discount on their next MDPI publication and can download a certificate of recognition directly from our submission system. Additionally, reviewers can sign up to the service Publons (https://publons.com) to receive recognition. Of course, in these initiatives we are careful not to compromise reviewer confidentiality. Many reviewers see their work as a voluntary and often unseen part of their role as researchers. We are grateful to the time reviewers donate to our journals and the contribution they make.

If you are interested in becoming a reviewer for *Sensors*, see the link at the bottom of the webpage http://www.mdpi.com/reviewers.

The following reviewed for *Sensors* in 2016:
Aalders, Maurice C. G.Ho, Megan Yi-PingPessin, GustavoAanstoos, JamesHo, MichaelPessoa, LuísAanstoos, James V.Ho, Siu WaiPetäjä, TuukkaAarts, Ronald M.Hoa, Suong V.Peteinatos, GerassimosAbbene, LeonardoHobbs, StephenPeter, MichaelAbd-Alhameed, RaedHoddle, MarkPeter, SteffenAbd-Elazim, S.M.Hodgson, LouisPeter, Van Der WalAbdelkefi, AbdessattarHoffman, Matthew J.Peters, KaraAbdelkefi, AbdessattarHöflinger, FabianPeterson, Donald R.Abdel-Motaleb, Ibrahim M.Holak, KrzysztofPeterson, Sean D.Abdel-Rahman, Mohammad J.Hölbl, MarkoPetit, LaurentAbdelsamea, MohammedHole, Lars R.Petit, NicolasAbdul-Aziz, AliHoller, StephenPetovello, MarkAbdulhalim, IbrahimHolmberg, Hans ChristerPetracca, MatteoAbe, HisashiHolzinger, AndreasPetriccione, MilenaAbidin, AysajanHomma, KazuakiPetro, Ana BelenAbot, Jandro L.Hong, DaesikPetroccia, RobertoAbouhossein, AlirezaHong, JinpyoPetros, BithasAbramovich, AmirHong, MenPetry, MarceloAbu-Nabah, BassamHong, MinPetsa, ElliAbuzneid, Abdel-ShakourHong, SeonkiPetti, DanielaAchterberg, Eric P.Hong, SungjunPettinato, SimoneAcuna-Bravo, WilberHong, SungkunPeyton Jones, JamesAdamos, Dimitrios A.Hong, SungwookPezoa, Jorge E.Addabbo, TommasoHong, Sun-MogPfammatter, AngelaAdebisi, BamideleHong, XiaopengPfeifer, ChristophAdeli-Mosabbeb, EhsanHong, YuningPfeiffer, ThiesAderohunmu, FemiHong, YuningPfletschinger, StephanAdewumi, AderemiHong, ZengPfletschinger, StephanAdibi, SasanHordacre, BrentonPflitsch, ChristianAdjizian, Jean-JosephHorgan, GrahamPham, CongducAdjrad, MounirHorng, Mong-FongPham, DucAfonso, José A.Horng, Shi-jinnPhinyomark, AngkoonAfseth, NilsHorns, DieterPhothisonothai, MontriAgafonov, VadimHorri, NadjimPhyo Wai, Aung AungAgarwal, MangilalHorrillo, María Del CarmenPianini, DaniloAghaei, Amirhossein ShokouhHorswill, Craig APiano, MartinaAghaie, Kiarash ZamaniHorvath, GyörgyPiao, XianjiAGOSTINI, VALENTINAHossain, A.K.M. MahtabPickering, MarkAgrafiotis, PanagiotisHostert, PatrickPicone, MarcoAgrawala, Ashok K.Hou, Shao YiPieczonka, LukaszAgüera-Vega, FranciscoHou, ShuangPieraccini, MassimilianoAgugiaro, GiorgioHouben, SebastianPieralice, FrancescaAgugliaro, Francisco ManzanoHoussineau, JeremiePierce, Leland E.Aguilar, ClaudioHoussineau, JeremiePierce Jr., Kenneth B.Aguilera, CristhiaHouston, JessicaPieri, FrancescoAguirre-Salado, Carlos A.Howard, Matthew J. W.Pierleoni, PaolaAhamed, JalalHowcroft, JenniferPieters, RoelAhamed, Mohammed JalalHoxley, DavidPietrabissa, AntonioAhlström, ChristerHoyhtya, MarkoPignaton De Freitas, EdisonAhmad, ZeeshanHrad, JaromirPiletsky, SergeyAhmadabadian, Ali HosseininavehHristoforou, EvangelosPillai, PrashantAhmadi, AminHsia, Kao-hsianPilloni, VirginiaAhmadi, AminHsiao, Chun-ChingPimenta, Tales CleberAhmed, KhandakarHsiao, Fu-yuenPimentel, Marco A.F.Ahmed, RiazHsiao, Hsu-ChunPina, Alberto BonastreAhmed, Syed HassanHsiao, Rong-ShuePinahasi, YosiAhmetovic, DraganHsiao, Shen-FuPinchin, JamesAhn, Hyeong-joonHsiao, VincentPineda-Sánchez, ManuelAhsant, BabakHsiao, Yung-ChiaPinho, PedroAiello, OrazioHsieh, Chih-ChengPinilla-Gil, EduardoAita, André LuizHsieh, KuangwenPinto, AntónioAkanyeti, OtarHsu, Chien-ChangPinto, LivioAkbaş, Mustafa İlhanHsu, Chih-shunPinto, RicardoAkcakaya, MehmetHsu, Heng-TungPinto, UelintonAketagawa, MasatoHsu, Huan-HsuanPipa, Daniel RodriguesAkhloufi, Moulay A.Hsu, Li-TaPipher, Judith L.Akhtar, ZahidHsu, Li-TaPiras, AndreaAkiba, ShigeyukiHsu, WensyangPiras, MarcoAkiba, YasutadaHsu, Yi-ChuPirozzi, SalvatoreAkins, Jonathan S.Hu, Can-MingPisco, MarcoAkitsu, TetsuyaHu, ChengchenPisello, Anna LauraAkkaya, IlgeHu, JiaPissadakis, StayrosAkkaya, KemalHu, Jia-ShengPita, MarcosAksanli, BarisHu, JinlianPittella, ErikaAksyuk, VladimirHu, NingPiva, StefanoAla, GuidoHu, NingPiyare, RajeevAlam, MuhammadHu, NingPłaczek, BartłomiejAlam, Muhammad MahtabHu, ShaohanPlasqui, GuyAlamán, XavierHu, XiaogongPławiak, PawełAlander, JarmoHu, XiaolingPleissner, DanielAlarifi, AbdulrahmanHu, XiuqingPlets, DavidAlba-Flores, RocioHu, ZhenxingPlyer, AurélienAlbajez, Jose AntonioHu, ZhongxuPo, Lai-ManAlbanese, DonatellaHua, XianghongPobelov, IlyaAlbano, MicheleHuang, Cheng-MingPoco, JorgeAlbarracín, RicardoHuang, Chih-ChingPodila, RamakrishnaAl-Bayati, AliHuang, Chih-WeiPoeggel, SvenAlbella, PabloHuang, Chih-YuanPoenar, Daniel P.Alberto, GrecoHuang, ChongPoggi, GiovanniAlberto, NeliaHuang, Genin GaryPohl, ChristineAlbiol, SergioHuang, Guang-BinPola, GiordanoAlbisetti, EdoardoHuang, JiadongPoland, JesseAlcock, LisaHuang, JianjunPoletkin, KirillAlcoutlabi, MatazHuang, Ji-JerPolitano, AntonioAl-Dahidi, SameerHuang, Jiung-yaoPollitz, FredAldea, EmanuelHuang, LeiPollock, BrandonAleixandre, ManuelHuang, Ming-ChunPolo, JoséAlejo, DavidHuang, PanfengPolygerinos, PanagiotisAleksandra, SimaHuang, PengchengPomalaza-Ráez, Carlos A.Alemán-Flores, MiguelHuang, QianPomares, Luis MartinAlenyà, GuillemHuang, Shih-ChangPomares Hernandez, Saul E.Alesanco, AlvaroHuang, Shih-ChiaPoncela, Dr. JavierAlessandro, CaporaliHuang, SunanPonsoda, EnriqueAlfano, GiusiHuang, WeiPoon, Chung Yan CarmenAlfinito, E.Huang, WenbinPoon, Chung Yan CarmenAlgarra, ManuelHuang, XinPoorter, Eli DeAlgorri, Jose FranciscoHuang, XingfengPopescu, MihailAli, AbdelrahmanHuang, XingjiuPopleteev, AndreiAli, MohammodHuang, YanboPopoola, OlalekanAli, MonsurHuang, YingPopovic, VladanAlian, Aymen A.Huang, Yo-PingPortaccio, MariannaAlim, SamatHuang, YulinPortalés, CristinaAlirezaei, GholamrezaHuang, Yung-FaPorter, ReidAl-Jaroodi, JameelaHuang, ZhiyaoPortet, FrançoisAllen, GrantHuang, ZufangPortilla, JorgeAlmalag, MohammadHuber, DanielPortilla, Jorge HigueraAlmasri, MahmoudHuber-Mörk, ReinholdPortugal, PauloAl-Masri, EyhabHübers, Heinz-wilhelmPost, Mark A.Almeida, AitorHuck, AlexanderPostalache, OctavianAlmeida, Fabrício C. L.Hudak, AndrewPostolache, OctavianAlmeida, Joao EmilioHudoklin, DomenPotortì, FrancescoAlmeida, JoseHugentobler, UrsPotter, John RAlmerico, Anna MariaHughes, RobPotter, MarkAlmond, Darryl P.Hugtenburg, RichardPottier, BernardAlmorox, JavierHui, GuoHuaPotyrailo, Radislav A.Alonso, Jesús B.Humenberger, MartinPoulakis, NikolaosAlonso-Gonzalez, AlbertoHumle, TatyanaPoulymenopoulou, MikaelaAl-Sarawi, Said F.Humphries, James R.Pound, MichaelAlshurafa, NabilHung, Ho-LungPoursanidis, DimitrisAltini, MarcoHung, Jui-chungPowell, RebeccaAltintas, ZeynepHung, TranPowers, DavidAlTunaiji, Ahmed Rashed KulaibHunt, AndrewPradalier, CédricÁlvarez Gil, María JoséHunt, HeatherPrager, JensAlves, HirleyHunt, Jr., E. RaymondPrasad, RangaRao VenkateshaAlves, MarioHuotari, MattiPrecup, Radu-EmilAlwan, EliasHur, JinPrelcic, Nuria GonzálezAl-Yasiri, AdilHur, KyeongPremaratne, PrashanAmal, HaithamHurni, KasparPrestininzi, AlbertoAman, NaveedHussein, MohamedPreuveneers, DavyAmanatiadis, AngelosHussein, Mohamed A.Priefer, RonnyAmante, BeatrizHuston, DryverPrieto Tejedor, JavierAmar, Achraf BenHutchins, DavidPrigozhin, GregoryAmatya, SurajHütt, ChristophPrinz, TorstenAmbellouis, SébastienHutu, FlorinProença, HugoAmditis, AngelosHuynh, Tan-PhatProrok, AmandaAmezcua Correa, RodrigoHwang, Chi-HungPrudenzano, FrancescoAmiri Moghadam, Amir AliHwang, Dong-HwanPryss, RüdigerAmiruzzaman, MdHwang, GilguengPrzybyt, MałgorzataAmmari, HabibHwang, Han-JeongPsaras, IoannisAmor, MercedesHwang, HoyoungPsimoulis, PanagiotisAmundarain, AiertHwang, Jenq-NengPu, FangAn, Min KyungHwang, Wen-JyiPu, HaihuiAn, NanHwang, Wen-LiangPu, LinaAnagnostopoulos, ChristosHyun, EuginPu, ShengliAnagnostopoulos, Christos B.Iacomi, FeliciaPucci, AnnemarieAnagnostou, Dimitris E.Iacono, MauroPugi, LucaAnantrasirichai, NantheeraIannacci, JacopoPuliafito, AntonioAnaya, José JavierIannizzotto, GiancarloPulli, KariAnceno, A. J.Iannizzotto, GiancarloPumera, MartinAndersen, Jens ChristianIanoul, A.I.Purdey, MalcolmAndersen, Kristoffer KIbarra-Castanedo, ClementePuttonen, EetuAnderson, ChristopherIbarra-Castanedo, ClementePyk, PawelAndrade, CarmenIbrahimi, KhalilPyun, Jae-youngAndré, PauloIbsen, MortenQi, JinAndré, PauloICKOWICZ, ADRIEN A.Qian, XuemingAndrea, GiantomassiIde, ReikoQian, YouAndreescu, SilvanaIdrees, HaroonQiao, DaYongAndreoni, GiuseppeIera, AntonioQiao, LeiAnfossi, LauraIgasaki, TomohikoQiao, LiAng, Li-minnIhalainen, PetriQiao, YuansongAngani, Chandra SekharIizuka, KojiroQiao, ZhijunAngani, Chandra SekharIles, RayQin, ChuanAngelidis, Pantelis A.Iliescu, CiprianQin, PeiyuanAnghel, AndreiIllarramendi, M. AsunciónQin, YanAngiolini, FedericoIllingworth, SamQin, YongruiAngrisano, AntonioIm, HyungsoonQin, YuanweiAngulo, CecilioImlau, MircoQin, ZhongyuanAnia-Castanon, Juan DiegoImtiaz, Syed AnasQiu, JianbinAnia-Castañon, Juan DiegoInamdar, AnandQiu, QiangAnishchenko, LesyaIndjin, DraganQiu, ShilunAnnamdas, VenuIndri, MarinaQiu, WeibaoAnnamdas, Venu GMIniesta, JesusQiu, ZhichengAnouar, BelahcenInomata, NaokiQu, HongweiAnsaloni, GiovanniInoue, JunichiQU, PENGAnsari, AzadehInvitto, SaraQu, YonghuaAnthony, CarlIoannides, NicholasQuan Qing, QuanAnthopoulos, LeonidasIoannidis, CharalabosQuattrini Li, AlbertoAntonopoulos, AngelosIoannis, KonidakisQuay, RayAntonopoulos, AngelosIodice, AntonioQueiruga-Dios, AraceliAntreich, FelixIonescu, AnisoaraQuero, GiuseppeAntunes, MichelIordachita, IulianQuerzoni, LeonardoAnvar, Amir MohammadIossifidis, IoannisQuinn, Mark KennethAnvar, Amir MohammadIreland, MurrayQureshi, AnjumAoki, HiroshiIsaacman, SibrenR. García, CarmeloAoki, KazuhiroIsaad, JalalRabbou, Mahmoud AbdAoyagi, TakahiroIsakov, DmitryRadac, Mircea-BogdanAoyagi, TakahiroIsalgue, AntonioRadecka, HannaAparicio, JoaquínIsern-González, JosepRadecki, JerzyAphale, Sumeet S.ISHIDA, ShigemiRadenkovic, MilenaApiecionek, ŁukaszIshikawa, JunRadke, KennethAppiah, KofiIshiyama, KazushiRadkowski, RafaelArafa, AhmedIshmanov, FarruhRadzik, TomaszArai, YasuoIslam, ShafiqulRahman, MasudurArami, ArashIslam, Syed KamrulRahman, ShahedurArana, SergioIslam, Syed KamrulRahmani, RahimArandjelović, OgnjenIsokoski, PoikaRahmatian, ArashAranha, JoseItahashi, SyuichiRajan, GinuArchambault, PhilippeIto, SoRallo, GiovanniArcher, NormIto, YosukeRalston, Jonathon C.Arcidiacono, ClaudiaIvanov, Ilia N.Ramahi, Omar M.Ardid, MiguelIvashov, SergeyRamalingam, RajinikumarArdila-Rey, Jotge AlfredoIwami, KentaroRamanavicius, ArunasArduini, FabianaIwamoto, MitsuguRamirez Lopez, Juan LeonardoArena, AntonellaIwasaki, AkiraRamírez Muñoz, DiegoArgenti, FabrizioIwata, TatsuyaRamirez-Paredes, Juan-PabloArges, ChristopherIyota, TaketoshiRamos, Helena GeirinhasArgüello, FranciscoIzquierdo, Joaquim BlesaRamos, IdaliaArgyriou, VasileiosJachowicz, RyszardRamos, VictorArias Hernández, Néstor AlonsoJackson, Glen P.Ramos, VictoriaAries, MyriamJackson, NathanRamos-Ortiz, GabrielAriga, KatsuhikoJacobs, PeterRampa, VittorioAriyur, KartikJafari, RezaRampazzi, SaraArkenbout, E. A.Jaffrezic-Renault, NicoleRanganathan, AanjhanArmada, ManuelJaffrezic-Renault, NicoleRangel-Magdaleno, Jose-de-JesusArmenta, SergioJaganathan, ArunRangelova, ElenaArmijo, FranciscoJahangiri, ArashRanger, Christopher M.Armstrong, JeanJaime, Castillo-LeónRantanen, VilleArmstrong, JeanJaimes, Luis GRao, AravindaArne, BröringJakiela, SlawomirRao, Posinasetti NageswaraArnó, JaumeJakóbczak, Dariusz JacekRao, SmithaArnold, F. J.Jakubik, Wiesław P.Raposo, MariaArnon, ShlomiJalal, AhmadRaptis, IoannisAroca, RafaelJamet, CédricRasilo, PaavoÁrpád, HuszákJamone, LorenzoRasmussen, Morten DamArregui, Francisco J.Jan, Shau-ShiunRatcliffe, NormanArribas, JavierJanegitz, Bruno CamposRathore, SaimaArriola, AitorJang, Byung-JunRaut, SangramArrue, Begoña C.Jang, Ho WonRaut, SangramArtese, GiuseppeJang, Jae EunRavani, BahramArtur, RydoszJang, Kyung-InRavelo, BlaiseArvaneh, MahnazJang, Yeong MinRawashdeh, Samir A.Arvapally, Ravi KumarJansen, BartRawassizadeh, RezaArya, Sunil KumarJansen, MarcusRawassizadeh, RezaAsadnia, MohsenJarabo-Amores, María PilarRawat, PriyankaAsakawa, NaokiJarchi, DelaramRay, LauraAsbeck, AlanJassim, SabahRayappan, John Bosco BalaguruAshok, AshwinJatana, Gurneesh S.Raymond, David R.Asorey-Cacheda, RafaelJavaid, Ahmad Y.Raynham, PeterAssante, DarioJavaid, NadeemRazaque, AbdulAsteriadis, Stylianos (Stelios)Javan-Khoshkholgh, AmirRazionale, Armando VivianoAstrain Escola, José JavierJavier Navarro, PedroRazzaque, M. A.Atchison, JenniferJaworek, AnatolReale, DiegoAthanasiou, LamprosJayamohan, HarikrishnanRebaudengo, MaurizioAtta, KhalidJayaraman, ChandrasekaranReboul, SergeAttarzadeh-Niaki, Seyed-HoseinJayaraman, PremRech, IvanAttivissimo, FilippoJayaratne, RohanReed, CarlAttux, RomisJe, ChangsooReed, NickAubin, PatrickJedelsky, JanReeves, Daniel BAugustyniak, PiotrJędrzejewska-Szczerska, MałgorzataRefice, AlbertoAull, Brian F.Jedrzejewski, Kazimierz PiotrRegazzoni, CarloAung, HaneJee, Gyu-InReggiani, Emanuele RobertoAuvinet, EdouardJegadeesan, RangarajanRegmi, BishnuAvanzi, FrancescoJekeli, ChristopherRegmi, Bishnu P.Avdikos, GeorgeJemielniak, KrzysztofRehmani, Mubashir HusainAvezov, EdwardJeng, Chyuan-hwanReig, CàndidAvolio, AlbertoJeng, Jyh-HorngReina-Tosina, Luis JavierAvram, MarioaraJenke, RobertReinoso, OscarAxenie, CristianJenkins, KennethReis, ArsénioAykin, Murat D.Jensen, Brian D.Reis, Manuel J.C.S.Azinheira, JoséJensen, Jesper RindomReisslein, MartinAziz, Md. AbdulJeon, GwanggilReju, V. G.Aziz, MuhammadJeon, MansikRekas, MieczyslawAzkune, GorkaJeong, Gu-minReljin, NatasaBabbar-Sebens, MeghnaJeong, Kyung JaeRemillieux, MarcelBabiarz, ArturJeong, Myeong-hunRen, BeibeiBaccaglini, EnricoJeong, Ok ChanRen, DahaiBacci, AlessioJeong, Taikyeong TedRen, HuazhongBachmann, JulienJeons, SangminRen, MingjunBackhouse, ChrisJha, SumitRen, MingwuBadilita, VladJi, Hong-bingRen, PinyiBadulin, SergeiJi, HongliRen, WeiBae, EuiwonJi, KefengRen, YongBae, Hyung DaeJi, LuyanRenaudin, ValérieBae, Ihn-hanJi, ShaoboRendón Mancha, Juan ManuelBae, YoungchulJi, WenRene, SergiBaeg, Seung-hoJi, YimingRenevey, PhilippeBaehr-Jones, TomJia, JiabinRenga, AlfredoBaek, StanleyJia, TonyRennie, MarkBaer, ChristopherJia, YunyiRenso, ChiaraBafekrpour, EhsanJia, YunyiRenzoni, FerruccioBaffou, GuillaumeJia, ZhenzhongReshetilov, Anatoly N.Bagci, UlasJiang, HaochuanResnick, AndrewBahillo, AlfonsoJiang, HongboRetscher, GüntherBai, HuaJiang, JinRetscher, GüntherBai, JinboJiang, Joe-AirRetterer, Scott T.Bai, JinheJiang, RuinianReverter, FerranBai, Jong-WhaJiang, ShanRevilla, EugenioBai, LuJiang, XiRevilla, PedroBai, XiangJiang, XiaoguangReyes, AngélicaBai, XueruJiang, XiaoningReyes, BersainBaik, Kwang HyeonJiang, XuchuanReyes, José SantosBailey, DonaldJiang, YifeiRezaei, MahdiBailón, RaquelJiao, LeiRhee, HuinamBaine, NicholasJiao, LihongRheem, JaeyeolBaiocchi, ValerioJiao, WeiliRhudy, MatthewBaker, Brendon M.Jiao, XianjunRhudy, Matthew B.Baker, RichardJimenez, FelipeRiahi, RezeBaker, TharJimenez, MelanieRiazuelo, LuisBakri-Kassem, MaherJimenez, RalphRibeiro, Adriana SantosBalandin, SergeyJimenez-Munoz, Juan-CarlosRibeiro, ÁngelaBalasingam, BalakumarJin, ChongjunRibeiro, Artur LopesBalbinot, AlexandreJin, HongxiaoRibeiro, EraldoBalda, MichaelJin, HosangRicci, StefanoBalkir, SinaJin, JiongRiccobene, GiorgioBallesta-Claver, JulioJin, Kyong HwanRichiedei, DarioBallestrin, JesusJin, MaolinRicotti, LeonardoBaltazart, VincentJin, MiaoRiedel, TillBamiedakis, NikosJin, NingdeRieger, RobertBamji, CyrusJin, QunRiliskis, LaurynasBanda, Juan M.Jin, Ren ChengRinaldi, FabioBanerjee, AshisJin, SunggeunRinaldi, StefanoBanerjee, TanviJin, TianRinaldi, StefanoBanishev, AlexandrJin, XiRinaldo, RobertoBanks, CraigJin, XinRinawan, Fedri RuluwedrataBañuls, Maria JoseJin, XueboRinge, EmilieBao, ForrestJin, YuanweiRipken, TammoBao, TengFeiJin, ZhanpengRishpon, JudithBaran, IsmetJin, ZhigangRitala, RistoBarański, PrzemysławJin, ZongwenRitcey, JamesBaratchi, MitraJING, JinRitchie, GlenBarbato, MarcoJo, JunRitchie, Matthew A.Barber, ZebJo, Kang-HyunRituerto, AlejandroBarberis, AntonioJo, MinhoRius, XavierBarbieri, GiuseppeJoe, Daniel J.Rivadeneyra, AlmudenaBarbosa, Carlos HallJohn, NielsenRivadeneyra-Torres, AlmudenaBarbosa, JoseJohnson, ChrisRiziotis, ChristosBarbosa, Ramiro S.Johnson, ColinRizzi, FrancescoBarceló, JaumeJohnson, DavidRizzoli, Andrea-EmilioBarille, RegisJohnson, Nigel P.Ro, RuyenBarlow, EuanJolani, FaridRoberts, DarBarniol, NuriaJong, Gwo-jiaRoberts, GethinBarnkob, RuneJoo, ChulminRocca, FabioBarolli, LeonardJoo, Ki-NamRocha, LuisBaron, ThomasJoo, Sang-WooRocha, Luís A.Barr, ChrisJoordens, MatthewRodenas-Herraiz, DavidBarralon, PierreJorge Ponzoni, FlávioRodger, James A.Barrera, FernandoJosé, Marcato JuniorRodriguez, MéndezBarrett, BrianJouanneau, SulivanRodriguez, NemesioBarrett, JohnJoubair, AhmedRodriguez, Sergio AlbertoBarrientos, AntonioJoubert, MichaelRodríguez, DionisioBarrientos Velazquez, Ana L.Joubert, StephanV.Rodríguez, FranciscoBarriga-Rivera, AlejandroJovic, MilicaRodríguez, Juan Manuel CorchadoBarron, JohnJozkow, GregorzRodríguez, Manuel DíazBarros, AntonioJozwik, MichalRodríguez, MarBarroso, JoãoJu, HeongkyuRodríguez, Roberto IglesiasBarroso, MargaridaJu, MinChulRodríguez Ángeles, AlejandroBarry, JonJuang, Jyh-ChingRodríguez Gonzálvez, PabloBarshan, BillurJung, AndrasRodríguez Martin, DanielBarsocchi, PaoloJung, CláudioRodríguez Méndez, María LuzBartelme, NorbertJung, Hyung-SupRodriguez-Falces, JavierBartelt, HartmutJung, JinhaRodríguez-Martín, ManuelBarth, RuudJung, Jung-YeulRodríguez-Molina, JesúsBartoletti, StefaniaJung, MinchaeRoemer, FlorianBartoli, GianniJung, Seong-OokRoerdink, MelvynBarzaghi, RiccardoJung, Suk-hoonRoger, LeblancBas, JoanJung, Young DoRogers, EricBas, Pierre-Yves LeJunker, Laura VerenaRogier, HendrikBasha, ElizabethJuránek, RomanRoh, JeongjinBasile, A.Jurkow, DominikRohac, JanBasit, Munshi MahbubulJurman, GiuseppeRohani, MohsenBastani, HamedJwo, Dah-JingRöhrig, ChristofBastawrous, HanyKaabouch, NaimaRohs, MichaelBasteris, AngeloKaasalainen, SannaRoig, IgnacioBastos, LuisaKacem, NajibRojas-Cessa, RobertoBasu, AnirbanKagawa, KeiichiroRolinski, OlafBaszynski, MarcinKagawa, MasaharuRoman, ChristopherBatalla, Jordi MongayKaimakamis, Evangelos K.Roman, RodrigoBatchelor, John C.Kaitovic, JelenaRomanato, FilippoBatista, Arnaldo GuimarãesKalantar Zadeh, KouroshRomano-Rodríguez, AlbertBatista, PedroKalayci, TunaRomero, GemaBaudoin, YvanKalchmair, StefanRomero, SergioBauer, MartinKalimuthu, PalrajRomero- Troncoso, ReneBauer, RomanKalivas, GrigoriosRomo Leon, Jose RaulBayford, Richard H.Kameoka, TakaharuRoncella, RiccardoBeales, Paul A.Kamienkowski, Juan EstebanRopars, ThomasBeauchamp, JonathanKamijo, ShunsukeRoriz, PauloBeben, DamianKampouris, DimitriosRos Lis, Jose VicenteBęben, AndrzejKamsu-Foguem, BernardRosa, StefanoBechelany, MikhaelKandris, DionisisRosado, Alfredo MuñozBechelany, MikhaelKang, ChulhunRosado, JaimeBechelany, MikhaelKang, DaeshikRoscia, MariacristinaBeckerle, PhilippKang, DongkyunRose, Joseph L.Beckers, KristianKang, DongyangRosenberg, LukeBedogni, LucaKang, HyunchulRosenberg, LukeBegoli, EdmonKang, InpilRosenstein, JacobBehar, JoachimKang, Je-WonRosolem, Joao BatistaBeheim, Glenn M.Kang, KyungranRossetti, RosaldoBehera, ArdhenduKang, Li-WeiRossi, AldoBein, DoinaKang, Min-WookRossi, René MichelBeisl, UlrichKang, PilgyuRossi, StefanoBéjar, RubénKang, Soon JuRossignol, JérômeBeko, MarkoKanjo, EimanRossomando, F. G.Bel, AlbertKanter, Theo G.Roth, BernhardBelavič, DarkoKanyong, ProsperRoth, Bradley J.Belikov, DmitryKapila, VikramRothkrantz, LeonBell, JesseKapuscinski, TomaszRoths, JohannesBell, MuyinatuKapuscinski, TomaszRothwell, Edward J.Bell, SimonKarabchevsky, AlinaRotroff, Daniel MillerBellavista, PaoloKaraiskos, GrigoriosRöttgers, RüdigerBelle, AshwinKarasik, BorisRoueff, AntoineBellido, Francisco J.Karcher, ChristianRoufarshbaf, HosseinBellone, MauroKarczmarek, PawełRouhani, HosseinBelmonte, OscarKarg, TimoRouhani, HosseinBeloglazova, N.Kari, ChadiRoupioz, YoannBelostotski, LeoKarim, Karim S.Rouse, Adam G.Belter, DominikKarimi, Hamid RezaRousseau, DavidBemmerer, DanielKarimi, HassanRousseau, LionelBen, YueyangKarimi, HassanRoussey, CatherineBenardos, PanoriosKark, LaurenRoveri, MarcoBenazouz, AymenKarkanis, Stavros A.Rovetta, StefanoBendahan, MarcKarlen, WalterRowlands, DavidBender, FlorianKarlgaard, Christopher D.Roy, CorinnaBender, PaulKarmakar, NemaiRoy, Raphaëlle N.Bendig, JulianeKarns, TaraRozen, OferBenedicenti, LuigiKartsakli, ElliRozga, AgataBenet Gilabert, GineKaruppiah, ChelladuraiRozhkova, ElenaBenetti, MassimilianoKarupppasamy, SubburajRuan, HaihuiBengtsson, LarsKaryotis, VasileiosRuan, HaowenBeni, V.Kasai, NaoyaRuano, AntónioBeni, ValerioKašalynas, IrmantasRucco, AlessandroBennetts, Victor HernandezKashani, Alireza G.Rückert, UlrichBenoussaad, MouradKasneci, EnkelejdaRückert, UlrichBenyahia, IlhamKasneci, EnkelejdaRudnitskaya, AlisaBen-Yoav, HadarKastner, RaphaelRudolph, HeikoBeongku, AnKatagiri, HiroshiRuff, AdrianBerberich, JasonKataoka, HirokatsuRuffier, FranckBerenguel, ManuelKataoka, MasatoshiRuffin, PaulBerenguer, RocKateris, DimitriosRufino, GiancarloBergaud, ChristianKato, FumitakeRuggeri, GiuseppeBergé, JoelKato, TeruyukiRühmer, DennisBerger, KaiKatsaros, KonstantinosRuiz-Hincapie, PaulaBerger-Vachon, ChristianKatsavounidis, IoannisRunnemalm, AnnaBergmann, Jeroen H. M.Katsura, SeiichiroRusso, MariateresaBergmann, NeilKaufmann, ViktorRusu, FlorinBergmann, Neil W.Kaur, AmandeepRuzzenenti, FrancoBergmann, OlafKaur, AmardeepRyoo, Young-JaeBeriáin, AndoniKauristie, KirstiRyu, DonghyeonBerkovic, GarryKaushik, AjeetSaal, HannesBernabe Garcia, SergioKavallieratou, ErginaSaavedra, Mario LilloBernacki, BruceKawalec, AdamSabatini, AngeloBernardi, GiulioKawano, TomonoriSabatini, RobertoBernas, MarcinKaya, TolgaSaccomandi, PaolaBerneschi, SimoneKazimierski, WitoldSachs, JuergenBernier, MartinKažys, Rymantas JonasSadjadi, FiroozBernier, MoniqueKe, YinghaiSadowski, ŁukaszBernini, RomeoKebkal, KonstantinSadr, AlirezaBerretti, StefanoKedzierski, MichalSaeed, KhalidBersani, MarcoKedzierski, MichalSagari, ShwetaBert, ChristophKehl, FlorianSaggio, GiovanniBerthoumieu, YannickKeikhosravy, KamyarSagha, HesamBertoncello, PaoloKeip, Marc-AndreSaghafi, BehrouzBertram, Timothy H.Kelly, KevinSagle, LauraBertsch, ArnaudKennet, K. OSahalos, John N.Besada, Juan A.Keogh, EamonnSaharan, LalitaBescós del Castillo, CristinaKeoh, Sye LoongSaharan, LalitaBestak, RobertKeshtkaran, Mohammad RezaSahay, PeeyushBétaille, DavidKeßler, CarstenSahay, PeeyushBettinger, PeteKeynton, RobertSahoo, JagrutiBevly, DavidKhalaf, WalaaSahoo, Prasan KumarBeyerer, JurgenKhalil Afzal, MuhamamdSahuquillo Balbuena, ElviraBeyerer, JürgenKhan, ImranSaijo, YoshifumiBezerianos, AnastasiosKhan, Jamil Y.Sainz, BeatrizBhagavatula, VijayakumarKhan, PervezSainz, Nekane IoneBhargava, SureshKhan, SaadSaito, KozoBhattacharjea, RajKhan, SaadSakagami, NorimitsuBhattacharyya, DebnathKhan, SaleemSakai, KazuyaBhattarai, KeshavKhan, Salman H.Sakamoto, João Marcos SalviBhojan, AnandKhan, Shehroz S.Sakar, Mahmut SelmanBhuiyan, Md Zakirul AlamKhan, UsmanSalama, SuhybBhuiyan, Md Zakirul AlamKhan, ZaheerSalamone, FrancescoBhuiyan, Sharif M. A.Khan, ZahoorSalanova Grau, Josep MariaBiagi, LudovicoKharkivskiy, SergiySalar-Garcia, M. J.Bian, ChaoKhatibi, Akbar A.Salazar-Torres, Jose J.Biancalana, ClaudioKhatibi, SiamakSaleem, KashifBianchi, MatteoKhmaladze, Alexander T.Salehi, BahramBianucci, PabloKhodabandeh, AmirSales, JorgeBicz, WiestawKhodaei, Zahra SharifSales, SalvadorBigall, NadjaKhoshelham, KouroshSaletti, RobertoBigaud, DavidKhoshnoud, FarbodSalgot, MiquelBigler, EmmanuelKhot, Lav R.Salido, DavidBilotta, EleonoraKiang, Jean-FuSalinas, YolandaBilstrup, UrbanKiani, MehdiSalleras, MarcBirch, BrianKiani Galoogahi, HamedSalm, CoraBird, JonKibangou, AlainSalmi, MikaBirlan, MirelKiełczyński, PiotrSalomoni, Sauro EmerickBisegna, FabioKieninger, JochenSalowitz, NathanBisio, IgorKikuchi, HiroakiSalvo Rossi, PierluigiBiswas, DwaipayanKim, AyoungSalz, PeterBiswas, PradiptaKim, Byeong-GyuSamczynski, PiotrBiswas, SubirKim, Byeong-WooSamek, WojciechBlair, StevenKim, Byung-SeoSamiappan, SathishkumarBlais, AntoineKim, Chang-SeiSamiei, EhsanBlakely, Jonathan N.Kim, Do YoungSampaio, SilvioBlanc, SaraKIM, Dong MinSampangi, Raghav V.Blanco, Jose LuisKim, Dong SubSampietro, DanieleBlas, Javier GarciaKim, DonghyunSampietro, DanieleBleakley, ChrisKim, Du YongSanakaran, SindhujaBleser, GabrieleKim, Eui-JikSanches, Ieda Del’ArcoBleser, GabrieleKim, Gil-HoSanchez, Jean-BaptisteBlock, StephanKim, Hae-JoSanchez, NancyBloisi, DomenicoKim, Hee-CheolSánchez, Joel Antonio TrejoBlume, HolgerKim, Hyug-HanSánchez de Rojas, José LuisBo, AIKim, Hyun JaeSanchez Soriano, Miguel AngelBoada, Beatriz L.Kim, HyunbumSánchez-Durán, Jose A.Boada, Maria Jesus LopezKim, HyunsungSanchez-Galan, JavierBobacka, JohanKim, Iltai (Isaac)Sanchez-Rodriguez, DavidBochenkov, Vladimir E.Kim, JaeyounSandoval Orozcoe, Ana LucilaBock, YehudaKim, JinsikSandrolini, LeonardoBoddington, RichardKim, Jin-WoongSanei, SaeidBodnar, Jean LucKim, Jin-YeonSanguesa, JulioBogaerts, JanKim, Jong SuengSangwine, S. J.Bogdan, PaulKim, Jong-HwanSanna, AndreaBoisseau, SebastienKim, JooheeSan-Segundo, RubénBoisselier, ÉlodieKim, Jung-SikSansone, LuciaBoissier, OlivierKim, KeonwookSantagata, AntonioBojic, IvaKim, KihwanSantamaria, CarlosBonaci, TamaraKim, Ki-hyungSantamaría-Gómez, AlvaroBonanno, GiovanniKim, KiilSantana, PedroBond, Tiziana C.Kim, Nam-YoungSantarato, GiovanniBondia, JorgeKim, Chang-SeokSante, Raffaella DiBonenberg, LukaszKim, Sang JaeSanthanagopalan, SunandBonev, Ilian A.Kim, Sang WooSanthiago, MuriloBonevich, John E.Kim, Sang WooSanti, FabrizioBoniface, KarenKim, Seon ManSantiago-Aviles, Jorge J.Bonin-Fon, FranciscoKim, SeungJunSantonico, MarcoBonod, NicolasKim, Seung-WooSantos, Auteliano ABoothe, TobiasKim, SoheeSantos, Davi AntônioBorboni, AlbertoKim, Sung KukSantos, LídiaBorecki, MichalKim, Sung-ManSantos, PauloBorghetti, BrettKim, Sung-PhilSantos Peñas, MatildeBorghi, AlessandraKim, SunwookSantulli, CarloBorgne, Hervé LeKim, Tae-seongSanyal, ArindamBorisov, SergeyKim, WonjunSanz, PedroBörner, AnkoKim, YongSappa, AngelBorri, SimoneKim, Yong-GooSappok, AlexanderBorschbach, MarkusKim, Young PilSarakis, LambrosBortot, ElianaKim, Young SikSareh, SinaBosch, ThierryKim, Young WookSarfraz, JawadBosiers, JanKim, Young-DukSarioglu, A. FatihBotella, GuillermoKim, YounghyunSarkar, SoumikBotella, GuillermoKim, Young-KeeSasagawa, KiyotakaBotha, Elizabeth JohannaKim, Young-KwanSasaki, KenBotterill, TomKim, YoungokSathyanarayana, SuchitraBotteron, CyrilKim, YoungwookSattler, TorstenBotzheim, JánosKinali-Dogan, AttilaSatzoda, RaviBouali, MarouanKinet, DamienSauermann, StefanBou-Cabo, ManuelKing, Matt A.Saukh, OlgaBouchard, KevinKinzel, EdwardSaunders, JeffBouk, Safdar HussainKiourti, AsiminaSauveron, DamienBoukallel, MehdiKira, ZsoltSavage, NickBourdon, PierreKirchner, JensSavazzi, StefanoBourtsoulatze, EirinaKiring, ArolandSavi, PatriziaBousquet, Jean-FrançoisKirk, RandolphSavin, IgorBoutaayamou, MohamedKirkko-Jaakkola, MarttiSavini, AntonioBoutayeb, HalimKirsanov, DmitrySavoia, AlessandroBouwmans, ThierryKissinger, DietmarSawacha, ZimiBouzefrane, SamiaKist, AlexanderSaxena, NavratiBouzid, Allal M.Kita, NobuyukiSaxena, NavratiBoverman, GregKitagawa, AkioSberveglieri, GiorgioBowles, Jeffrey H.Kitani, TomoyaSberveglieri, VeronicaBoyd, AnthonyKitano, ClaudioSberveglieri, VeronicaBoytsov, AndreyKittas, ConstantinosScalzo, FabienBozkurt, AlperKjaer, KatrineScapaticci, RosaBozzano, RobertoKjelstrup, SigneScappatici, LorenzoBrabec, MarekKlaoudatou, EleniScarpiniti, MicheleBraca, PaoloKlein, ChristianSchaefer, Hans-EckhardtBračun, DragoKlein, JuliusSchagaev, IgorBraga, Guilherme S.Klempous, RyszardSchall, Mark C.Braga, Roberto AlvesKlenk, JochenSchatz, Bruce R.Bragos, RamonKlenk, JochenSchauer, ThomasBranas, ChristianKlepka, AndrzejSchellen, H.l.Brandao, Alexandre SantosKlingbeil, LasseSchena, EmlianoBrandao, PedroKlueh, UlrikeSchenato, LucaBrandl, M.Klueh, UlrikeSchiavulli, DomenicoBrando, CarmenKo, Byoung ChulSchiavulli, DomenicoBranson, David T.Ko, Chia-NanSchilling, MalteBrante, GlauberKo, ConnieSchilling, MeinhardBratov, AndreyKo, HoonSchindelhauer, ChristianBräuer-Burchardt, ChristianKo, HyounghoSchirhagl, RomanaBraun, AlexanderKo, Kwang HeeSchlanbusch, RuneBraun, AndreasKo, Nak YongSchlegel, Joshua P.Brayner, AngeloKobayashi, MakikoSchloss, PatrickBrazhe, NadezdaKobayashi, ShunsukeSchmidt, AdamBreaker, RonaldKoch, ChristianSchmitt, CorinnaBreckon, TobyKociuba, WaldemarSchmitt, CorinnaBreier, J. A.Koehler, KirstenSchmitt, MartinBreitkreutz, JörgKoenig, KeithSchmitt, MichaelBrennan, Robert W.Koh, JaehanSchmitt, RobertBretterklieber, ThomasKoh, Kah HowSchmitz, AlexanderBreuer, StefanKoh, Kim EanSchneckenburger, HerbertBreunig, IngoKohl, Paul A.SchneeklothJr., John S.Brie, DavidKohut, PiotrSchneider, ThomasBrigham, KatharineKoinkar, PankajSchnitman, LeizerBriguglio, NicolaKõiva, RistoSchoedel, AlexanderBrimblecombe, PeterKojima, FumihideSchoening, TimmBrischke, ChristianKokkinis, GeorgiosScholefield, AdamBrisolara, Lisane Brisolara DeKokkinos, ChristosScholten, KeeBrito, PauloKokkinos, PanagiotisScholz, MarkusBrodie, Matthew A.Kołakowska, AgataScholz, MiklasBrolly, MatthewKolakowski, JerzySchön, SteffenBrolly, MatthewKolpashchikov, DmitrySchöps, SebastianBromaghin, Jeffrey FKomarova, Natalia V.Schotte, KenBronzi, DaniloKomnios, IoannisSchotter, JörgBrosed, Francisco JavierKompatsiaris, IoannisSchrader, AndreasBrosseau, ChristianKong, DemingSchrama, Ernst J.O.Broumandan, AliKong, JinmingSchroeder, WilfridBrouzes, EricKong, L.B.Schühle, UdoBrouzgou, ΑngelikiKong, Siu-KaiSchulman, RebeccaBrown, Laura C.Kong, WanzengSchultz, StephenBrown, StephenKong, XiangjieSchulz, RuthBrown, Tim Wc CKongsro, JørgenSchulz, UlrikeBrown, TimothyKonh, BardiaSchumacher, JensBrownlee, SandyKönig, PeterSchumann, HenrikBrückl, HubertKonovaltsev, AndriySchumm, BenjaminBruder, StephenKonrad, JanuszSchüßler, MartinBruggemann, TroyKonstantinos, ChousianitisSchütze, AndreasBruggi, MatteoKonstantinou, NikolaosSchwartzman, David JohnBrujic-Okretic, VesnaKonstantopoulos, CharalamposSchwind, PeterBrunelli, DavideKoo, ChiwanSchwotzer, MatthiasBrunner, Andreas J.Kooistra, LammertSciarrone, AndreaBrunner, PeterKopacek, PeterScilingo, Enzo PasqualeBruno, FabioKopackova, VeronikaScioscia, FlorianoBruno, FabioKopeika, NatanScognamiglio, VivianaBruno, Thomas J.Kopel, PavelScotney, BryanBruschetta, MattiaKopelevich, OlegScott, Jonathan BBruschi, PaoloKoppal, SanjeevScott, TomBruschini, ClaudioKopra, KariScowen, PaulBrust, Matthias R.Korets, MikhailScully, Christopher G.Bryan, PaulKörner, MarcoSebök, ÉvaBrydegaard, MikkelKorostynska, OlgaSecco, EmanueleBryson, M.Korposh, SerhiySeco-Granados, GonzaloBrzozowska, EwaKowalski, Piotr A.Sedlmeir, FlorianBuchmann, ErikKos, AntonSee, Chan H.Budianu, AlexandruKosa, GaborSee, Khay WaiBudzier, HelmutKosarev, AndreySeel, ThomasBueno-Martinez, AntonioKosinka, JiriSeet, Boon-ChongBugdol, MarcinKoskela, MarkusSegawa, HiroyoBuhot, ArnaudKosmala, MargaretSeginer, IdoBui, Minh-PhuongKosmopoulos, DimitriosSeiner, Brienne N.Bui, NicolaKostamovaara, JuhaSeitz, JochenBujari, ArmirKostopoulos, VassilisSeitz, PeterBulbul, AbdullahKothapalli, Sri-RajasekharSekercioglu, AhmetBuonocore, FrancescoKothe, SteffenSelleri, StefanoBurger, ThomasKotsiantis, SotirisSelvaraja, Shankar KumarBurian, AdinaKotsifakos, AlexiosSelyanchyn, RomanBurleigh, ScottKottapalli, Ajay Giri PrakashSemmar, NadjidBurrascano, PietroKottege, NavindaSencan, IkbalBurri, SamuelKotthaus, SimoneSendra, SandraBurrows, SusanKotze, BenSendra Compte, SandraBuscaglia, MarcoKoubias, StavrosSenesky, Debbie G.Bush, Brian WKoulamas, ChristosSengul, CigdemButt, HaiderKoumoulos, Elias P.Sengupta, AnupamByl, Nancy NKourgialas, NektariosSengupta, BidishaByrne, HughKourkouli, PenelopeSeo, Bo-SeokByrne, RobertKoutsias, NikolaosSeo, Gab-SuByun, HeejungKoutsoudis, AnestisSeo, Min-WoongCataldo, Andrea Kouvelas, AnastasiosSeo, SungkyuCaballero, PinoKouzoudis, DimitrisSeppanen, T.Caballero-Gil, CándidoKovalev, SergeySeppänen, AkuCaballero-Gil, PinoKozlowski, LesterSequeira, JoãoCabanes, GuenaelKraft, Reuben H.Serena, MorigiCabeza, RafaelKrasnov, Oleg A.Serôdio, CarlosCabrera, Juan AntonioKrause, André FrankSeron, DavidCabrita, M. J.Krause, Hans-JoachimSerra, GiuseppeCaciolli, AntonioKrause, ThomasSerrano, José IgnacioCahalane, ConorKrehel, MarekSerrano, Mario EmanuelCahoy, KerriKrejcar, OndrejSerry, MohamedCai, WenruiKrejcar, OndrejServin, ManuelCai, YinghaoKremens, BobSessa, SalvatoreCai, ZhipengKrief, FrancineSeveri, StefanoCai, ZhipengKrijnen, B.Severino, RicardoCain, StephenKrishnan, PraveenaSeviour, ThomasCaizzone, StefanoKristoffersson, AnnicaSeyler, FrédériqueCajal, CarlosKrólak, AleksandraSezgin, AydinCakmak, OnurKrólicka, AgnieszkaSha, KeweiCalamita, GiuseppeKrommenacker, NicolasShafiee, HadiCalbimonte, Jean-PaulKronberger, RainerShafiei, MahnazCalcante, AldoKropatsch, WalterShah, LawrenceCaldas, PauloKropfreiter, ThomasShahbazi, MahmoudCaldas, PauloKrüger, BjörnShaheen Elgamel, MohamedCaldeira, João M. L. P.Krull, Ulrich J.Shahmohammadi, MohsenCaldeira, Joao Manuel L. P.Krusienski, DeanShahrabi-Farahani, AlirezaCaleffi, MarcelloKruss, SebastianShahzad, AamirCalli, BerkKryjak, TomaszShaker, GeorgeCallico, Gustavo M.Krzeszowski, TomaszShalaby, MostafaCalonico, DavideKsentini, AdlenShamsi, Mohtashim HassanCalveras, AnnaKsiezopolski, BogdanShan, HangguanCalvo Rolle, Jose LuisKuang, QinShan, Xiaojun (Gene)Cam, HasanKuang, ShiboSHANG, FangCamarinha-Matos, Luis M.Kubo, IzumiShang, JiangaCamas-Anzueto, J.L.Kuc, RomanShang, YuCampatelli, GianniKuc, Tae-YongShangguan, LongfeiCampbell, CarleneKudela, PawełShankar, VenkyCampo, EricKudo, MineichiShapter, Joe G.Campos, InmaculadaKugiumtzis, DimitrisSharma, MrigankCampos, RicardKuipers, BenjaminSharma, Piyush SindhuCampos Rubio, Juan CarlosKulathumani, VinodSharma, RajnikantCampuzano Ruiz, SusanaKulatunga, ChamilShe, YuCan, ZuhalKulesza, WlodekShegai, TimurCanete, FranciscoKulikova, MariaSheinker, ArieCannon, BretKull, ChristianShekhawat, GajendraCano Garcia, Jose ManuelKulpa, KrzysztofShen, HuanfengCantley, Kurtis D.Kulshreshth, ArunShen, HuiliangCao, HongruiKumada, AkikoShen, Tsu-WangCao, HungKumar, ManishShen, WenCao, Jian-nongKumar, PankajShen, YaochunCao, LeiKumar, PardeepShen, YiranCao, WeiKumar, PardeepSheng, BinCao, XianghuiKumar, PardeepSheng, DuoCao, XinKumar, Suryadevara NagenderSheng, WenyiCao, YueKumar, VimalSheng, ZhengguoCao, ZongjieKumar, VivekShepherd, PeterCao-Paz, Ana MaríaKumar Somappa, Admar AjithSheppard, JamesCapella, Juan VicenteKumon, MakotoSheta, BassemCapilla, RafaelKuo, Chao-LinShevtsov, SergeyCapineri, LorenzoKuo, Chih-YuShi, BoxinCAPPA, PaoloKuo, Chung-HsienShi, FeiCapuano, RosamariaKuo, Chung-YenShi, KeCaputo, DomenicoKuo, WatsonShi, QihuiCarbonaro, NicolaKuo, Ye-ShengShi, TiezhuCarbone, GiuseppeKuremoto, TakashiShi, YangCarboni, MicheleKurenda, AndrzejShi, ZhanCardellach, EstelKurillo, GregorijSHI, ZUOQIANGCardenas-Juarez, MarcoKurlyandskaya, GalinaShiang, Tzyy-YuangCardinaels, RuthKuroda, RihitoShibata, NaokiCardoso, José RobertoKurokawa, SyuheiShieh, Wann-YunCardoso, SusanaKurt, MehmetShih, Ching-longCarlen, Edwin T.Kurt, MehmetShih, Kuei-PingCarli, RuggeroKurz, FranzShih, SteveCarlsson, MarcusKusche, JürgenShih, Wen-PinCarminati, MarcoKushki, AzadehShikida, MitsuhiroCarmo, JoãoKussul, NataliiaShiku, HitoshiCarmona, Pedro LatorreKusyk, JanuszShim, Duk-SunCarmona-Murillo, JavierKuzmanic Skelin, AnaShim, Yoon-BoCarolis, Berardina DeKwak, JeonghoShin, Bok-SukCarrascosa, Laura GarciaKwak, Moon K.Shin, Dong JinCarter, W. E.Kwak, YunsikShin, HainsworthCartiaux, OlivierKwok, Ngai MingShin, HyuncholCaruso, GerardoKwon, Jae KyunShin, JitaeCarvajal, RodrigoKwon, Jae KyunShin, MinhoCarvajal Rodríguez, Miguel AngelKwon, Jay HyounShin, Seung WookCarvajal-Ramirez, FernandoKwon, Oh-KyongShin, Soo-YoungCarvalho, HelderLa, Hung (Jim)Shintaku, HirofumiCarvalho, LidiaLa Forgia, NicolasShojafar, MohammadCasals-Terré, JasminaLabati, Ruggero DonidaShojafar, MohammadCasas, Joan R.Labib, MahmoudShoji, YoshinoriCasas, OscarLachapelle, GérardShoshany, MaximCasciati, FabioLad, Robert. JShrestha, Lok KumarCasilari Pérez, EduardoLaflamme, SimonShrestha, MaheshwarCasimiro, AntónioLagarde, FlorenceShu, XuewenCasimiro, TeresaLagkas, ThomasShui, LinglingCasquel, RafaelLago, Claudimir Lucio DoShukla, Sudheesh K.Cassara, PietroLagouarde, Jean-pierreShull, Peter B.Cassioli, DajanaLagüela, SusanaShum, Hubert P. H.Casson, AlexLahoz, William A.Shuman, VeraCastagnetti, CristinaLai, FeipeiSi, PengCastanon, DavidLai, PuxiangSianov, AnatoliiCastellani, FrancescoLai, Ying-ChihSidiropoulos, PanagiotisCastilla, GuillermoLai, Yu-KunSiebert, J. PaulCastillo, CarlosLakakis, KonstantinosSignore, GiovanniCastillo Sequera, José LuisLakard, BorisSikora, MarekCastro, Maria Claudia FerrariLalos, Aris SSikorski, WojciechCastro, MickaëlLAM, Kwok-hoSilla, CarlosCatalin, AlexandruLam, Raymond H. W.Silva, CarlaCataliotti, AntonioLamb, DavidSilva, Everson Thiago Santos Gerôncio DaCattaneo, Marco E. G. V.Lamberti, FabrizioSilva, IvanovitchCattelani, LucaLami, IhsanSilva, LígiaCavagnaro, MartaLammens, NicolasSilva, Rone Ilídio DaCavalcante, Charles CasimiroLamonaca, F.Silver, JasonCawkwell, FionaLamouroux, EmmanuelSilvester, Debbie S.Cazorla, MiguelLamparelli, Rubens Augusto CamargoSilvestri, LeonardoCazzulani, GabrieleLan, Kun-ChanSim, Chow-Yen-DesmondCegla, FredericLan Jiang, LanSim, Sung-HanCelesti, AntonioLancaster, DavidSima, ChaotanCella, LauraLandaluce, HugoSimeu-Abazi, ZinebCelli, AnnaLandes, T.Simic, MilanCempini, MarcoLaneve, GiovanniSimic, MilanCena, GianlucaLanfranchi, VincentSimoens, FrancoisCennamo, NunzioLang, JeffreySimon, LaurentCenteno, AnthonyLang, JochenSimone, AndreaCentonze, DiegoLangmans, JelleSimonian, Aleksander L.Cepuder, PeterLangston, CharlesSimos, PanagiotisCeravolo, RosarioLanitis, AndreasSinclair, Anthony N.ČernÝ, MartinLantada, Andres DiazSingelée, DaveCervantes, JairLantieri, ClaudioSingh, AdityaCesano, FedericoLantos, BélaSingh, AsheeshCetin, EdizLantsoght, EvaSingh, Atul KumarCha, Young-JinLanza-Gutierrez, Jose M.Singh, DhananjayCha, YoungsuLanzola, GiordanoSingh, DhananjayChaaraoui, Alexandros AndréLaopoulos, TheodoreSingh, Kamal DeepChadli, MohammedLapington, JonSingh, RanjanChady, TomaszLappakoski, HelenaSingh, RiturajChah, KarimaLargillier, ThomasSipal, VitChahl, JavaanLari, ZahraŠípová, HanaChahuara, PedroLarijani, HadiSiqueira, AdrianoChai, RifaiLarijani, HadiSiqueira Dias, José A.Chakraborty, SubhadeepLarrarte, FrédériqueSirmacek, BerilChalioris, Constantin Ε.Larson, EricSirohi, JayantChalopin, ClaireLaski, PawelSisinni, EmillianoChalvatzaki, GeorgiaLasky, TySismaet, HunterChambers, JonathonLasri, TuamiSisniega, AlejandroChan, AndrewLatella, ClaudiaSitnik, RobertChan, Chia-TaiLattuada, MarcoSiu, Kin Wai MichaelChan, Eddie C. L.Latychevskaia, TatianaSjögren, ThomasChan, GaryLau, LawrenceSkakun, SergiiChan, Kit YanLau, MichaelSkierucha, Wojciech M.Chan, Leanne Lai-HangLaurenzi, SusannaSkjelvareid, Martin HansenChan, Pak KwongLavagno, LucianoSkjetne, RogerChan, Yu-WeiLavoie, BenjaminSkonieczny, KrzysztofChanda, EmmanuelLay-Ekuakille, AiméSkouby, Knud ErikChandrahalim, HengkyLay-Ekuakille, AiméSkubic, MarjorieChanel, GuillaumeLazar, JosefSkyllas-Kazacos, MariaChang, C.C.Lazarescu, Mihai TSloan, RobinChang, Chih-YuanLazaro, AntonioSlobodian, PetrChang, Chi-jungLázaro, JesúsSmet, MarioChang, ElizabethLázaro, José L.Smeu, EmilChang, En-ChihLázaro, María TeresaŠmíd, RadislavChang, Herng-huaLazzeri, RobertaSmietana, MateuszChang, HonglongLe, Duc VanSmirnov, AlexanderChang, Hung-WeiLe, QiangSmith, BenjaminChang, IPLe Bars, FabriceSmith, BethChang, JhihchungLe Meins, Jean-FrançoisSmith, David B.Chang, JingboLe Pioufle, BrunoSmith, EmilyChang, Kang-MingLea, RodgerSmith, Lloyd M.Chang, Ming-WeiLeach, MarkSmith, StephenChang, Pei-ZenLeadbetter, AdamSmith, StewartChang, Ray-iLeccese, FabioSmolka, JacekChang, Rwei-ChingLeccese, FabioSmolyaninov, IgorChang, S.Y.Lee, Bih-HwangSmulko, JanuszChang, Shao-HsiaLee, BoheeSmyth, TimChang, Sheng-PoLee, Byeong HaSnape, JamieChang, Suk TaiLee, Chang-SeopSo, JaewooChang, Tae GyuLee, ChangwonSoar, JeffreyChang, Tong-BouLee, Chau-hwangSoares, Vasco N.G.J.Chang, Yao-JenLee, Cheng-ChiSochol, RyanChang, Yau-ZenLee, Cheng-ChiSöderholm, StefanChang, Yia-ChungLee, Cheng-LingSöffker, DirkChang, Yuan-JenLee, Chien-NanSoh, Gim SongChang, YuchunLee, Chi-YuanSohgawa, MasayukiCharbon, EdoardoLee, Dae-SikSohi, Ali NajafiChardon, GillesLee, Dah JyeSojic, AleksandraCharleston-Villalobos, SoniaLee, Da-shengSolari, FabioCharlton, Peter ChristopherLee, DonghwaSolbach, KlausChatzi, EleniLee, DonghwaSoleimani, ManuchehrChau, K.W.Lee, Edward A.Soliman, Abdel-HamidChau, Lai-KwanLee, Gil-YongSolimene, RaffaeleChau, YuenLee, Gim HeeSolle, DörteChaudhary, UjwalLee, Gyu MyoungSoltanalian, MojtabaChávez, EstebanLee, Han-Bo-RamSon, HungsunCHE, WeitaoLee, HeesungSon, JunggabChebat, Daniel-RobertLee, Hsiang-ChiehSonato, AgneseChegel, VladimirLee, Hsiao-YiSONG, AiguoCheikh, Faouzi AlayaLee, Hung-KyuSong, EdwardChellappa, RamaLee, Hyang-WonSong, HoubingChen, BoLee, Hyeong JaeSong, JifengChen, Bor-SenLee, HyokiSong, KaiChen, CailianLee, HyokiSong, KaichenChen, Chang-HsiaoLee, HyukjinSong, MinkyuChen, Chao Yu PeterLee, HyungJuneSong, Taek LyulChen, Chaur-TzuhnLee, Il-GuSong, TianChen, ChenLee, Jae HoonSong, Xiao-PengChen, Chiao-EnLee, Jae YoungSong, Yong-Ak (Rafael)Chen, Chien-FuLee, JangmyungSong, Yong-HaChen, Chi-HuaLee, JejungSong, Young-JoonChen, Chuh-YungLee, Jeong SuSong, YuChen, Chuin-ShanLee, Jeong-SooSong, ZhanChen, Chung-HaoLee, Jong-HeunSoon, Yeo TatChen, Chyong-HuaLee, Jong-HoSoong, Boon HeeChen, DeJiuLee, JoshuaSørensen, Anders StengaardChen, Denis GuangyinLee, Jung KeunSorge, ChristophChen, Der-chinLee, JungchulSoria, SilviaChen, GangLee, KeekeunSoriano, Julio GómezChen, HaimingLee, Ki YongSoriano, LauraChen, HaiyanLee, Ki-WonSorli, Bricechen, HaoyaoLee, Kuan-HuiSorrentino, MarcoChen, HongLee, Kuan-HuiSośnica, KrzysztofChen, HonglongLee, Milton L.Sossa Azuela, Juan HumbertoChen, HongyangLee, Myung-JaeSoto, IgnacioChen, HuifangLee, Peter Peng FooSoua, RidhaChen, HuilinLee, S.Soylu, AhmetChen, JiaLee, Sang-GeolSpaccini, DanieleChen, JiajunLee, Sang-GonSpachos, PetrosChen, JieLee, Seok JaeSpagnolo, VincenzoChen, JunpingLee, Seung-WooSpagnolo, VincenzoChen, Jyh-chengLee, Soo YeolŠpakov, OlegChen, KongzheLee, Suk JinSpalletti, AnnaChen, LiangLee, SungyoungSpampinato, ConcettoChen, LiangliangLee, Tae-EogSpanakis, Emmanouil GChen, Lien-WuLee, TaekkyeunSpanos, Costas J.Chen, LingxinLee, TaikjinSparavigna, Amelia CarolinaChen, Lung-ChienLee, Tian-FuSparkes, ValerieChen, MinLee, Tzung-LinSparks, RossChen, MinLee, Won GuSpecht, CezaryChen, MouLee, Woo-KyungSpedicato, LuigiChen, NanLee, WooyoungSpeliotis, ThanassisChen, NengchengLee, YangSpenza, DoraChen, Pai-YenLee, Yang-hanSpies, FrançoisChen, PeirongLee, Yong WookSpilka, JiříChen, Po-YuLee, Yong-hanSpinsante, SusannaChen, QiangLees, John E.Spivak, ArthurChen, QuanshengLefeuvre, ElieSpivak, ArthurChen, ShanbenLeger, Jean-MichelSpolaor, FabiolaChen, Shih-JuiLegg, MathewSporea, DanChen, Shyi-MingLehtola, VilleSpreer, WolframChen, SiluLei, ChongSpriet, CorentinChen, SiweiLei, JianpingSprint, Gina L.Chen, Tseng-YiLei, TingWuSprlak, MichalChen, WeiLei, YaGuoSpüler, MartinChen, WuLei, YangSquella, J.A.Chen, WujunLeitao, Diana C.Sraj, IhabChen, XianfengLeitis, KarstenSreenivas, Ramavarapu S.Chen, XiaojiangLekawa-Raus, AgnieszkaSridharan, D.Chen, XiaoqianLekkala, JukkaSrinivasan, GopalanChen, XueminLemaire, Martin T.Stadler, BethanieChen, Yen-DaLEMEUNE, AllaStamos, IoannisChen, Yen-LinLemoyne, RobertStankovic, LinaChen, Yi-ChaoLengden, MichaelStankovic, LjubisaChen, Yi-ChenLengfeld, KatharinaStanwix, PaulChen, YongyaoLensch, HendrikStarecki, TomaszChen, Yuan-TsungLent, RicardoStarkey, DakotaChen, YuanzhuLenzen, ChristophStarobin, Joseph M.Chen, YueLeon, CarlosStaruch, MargoChen, Yu-HsuanLeone, AlessandroStassi, StefanoChen, ZhigangLeone, PierreStaszewski, Wieslaw JChen, ZonghaiLeong, ThomasStavrakoudis, Dimitris G.Cheneler, DavidLerasle, FrédéricSTAVROS, IEZEKIELCheng, Chia-ChiLerchner, JohannesStavroulakis, Georgios E.Cheng, Chia-HsinLeroux, PaulStech, JoseCheng, Chien-FuLesch, AndreasStechele, WalterCheng, Ching-HsiangLesiak, PiotrStefani, AlessioCheng, Chi-TsunLetia, TiberiuStefani, AlessioCheng, FangLeung, Christopher K. YStefano, Luca DeCheng, GongLeung, Chung-HangSteffen, HolgerCheng, JingyuanLeung, YeeSteinberg, Steven J.Cheng, JuanLeutenegger, MarcelSteinhauer, StephanCheng, Leo K.Leverington, David W.Stelmashuk, VitaliyCheng, Marvin H.Levesque, VincentStephani, HenrikeCheng, PengLevi, AlessandroStergiou, DimitrisCheng, Shin-MingLevia, Delphis F.Sternberg, Ben K.Cheng, ShunfengLevy, IlanStevanovic, AleksandarCheng, WeiLey-Cooper, YusenSteven, AndyCheng, Wei ChenLeyk, StefanStevens, ChrisCheng, Wood-hiLezoray, OlivierStevens, EricCheng, XiuzhenLi, AihuaStevens, MatthiasCheng, Yu JianLi, AinongStevens-Navarro, EnriqueCheng, YuanLi, BaoxinStewart, ArthurCheng, ZhiqiangLi, BingStirling, LeiaCheong, Jia HaoLi, ChongStiros, StathisChesmore, DavidLi, Chun-HsingStobiecka, MagdalenaCheung, GeneLi, DerenStocker, MarkusChevalier, AlexisLi, DeshiStoffregen, ThomasChevalier, Francois LeLi, FanStone, ErikChiang, Chia-ChinLi, FangStone, Kenneth CChiang, Kai-WeiLi, FengStopforth, RiaanChiang, Kuo-NingLi, GangStoppa, DavidChiang, Ming-HsiLi, HaiyanStoppini, AurelioChianucci, FrancescoLi, HaiyangStork, WilhelmChiarenza, GiuseppeLi, HaoStoytcheva, MargaritaChiavaioli, FrancescoLi, HongguangStrakowski, MarcinChien, Ying-RenLi, HuanStrąkowski, MarcinChih, MingchangLi, JiaStrangi, GiuseppeChih, Ming-YuanLi, JianStrauss, AlfredChilo, JoséLi, JianStrehlitz, BeateChin, Cheng SiongLi, JichaoStrelcov, EvgheniChin, Lip KetLi, KaiStrittmatter, AndréChing, Congo Tak ShingLi, LiangStrohm, EricChiolerio, AlessandroLi, LinStruk, PrzemyslawChiou, Jin-ChernLi, LinStruk, PrzemyslawChirizzi, DanielaLi, MeixianStrumiłło, PawełChisum, JonathanLi, MinStruthers, Allan A.Chiu, Chen-WeiLi, MoStumm, ElenaChiu, Tai-chiaLi, Paul C.H.Sturtevant, BlakeChiu, YiLi, PeijunSu, Ching-liangChiva, SergioLi, PeiwuSu, Chun-HsuCho, BeongkiLi, Peter ZhixiongSu, FulinCho, Dongil DanLi, QingguoSu, Kuo-LanCho, Eun JeongLi, RenfaSu, Ming-ShouCho, GihwanLi, TianchengSu, Mu-chunCho, HansangLi, TianchengSu, Pi-GueyCho, Ho-ShinLi, TonglinSu, RuoyuCho, SoojinLi, WenjiaSu, ShaoCho, Sung HoLi, WenjuanSu, Yu-FengCho, YounghoLi, WenliangSu, ZhongChodavarapu, VamsyLi, WenlingSuarez, AlvaroChoi, Byong-DeokLi, XiaoliSuárez Ruiz, FranciscoChoi, Cheon WonLi, XiaopengSugano, MasashiChoi, Han-LimLi, XimingSuh, Doug YoungChoi, HojongLi, XingxingSuh, Young SooChoi, Hyo-JickLi, XingxingSuhling, KlausChoi, Hyun-HoLi, XinwuSujit, P.Choi, In-SikLi, XuejinSulla-Menashe, DamienChoi, Jae-WonLi, YangSullivan, Pierre E.Choi, Jae-WonLi, YangminSullivan, Rani W.Choi, Jeong WooLi, YingguangSumetsky, MichaelChoi, Ji-WoongLi, YizhenSun, EnjiChoi, Joon-HoLi, YongSun, GuanghuiChoi, JunilLi, YongSun, HaoChoi, JunilLi, YouSun, Jia-HongChoi, Jun-WonLi, YundongSun, JiguangChoi, KyusunLi, YuweiSun, JinweiChoi, MinhaLi, ZhanSun, LiangChoi, Sei-BumLi, ZhenjiangSun, LuniChoi, SeokheunLi, ZhiSun, MengChoi, Seon-JinLi, ZhiwuSun, Nian X.Choi, Woo JuneLI, ZhongliangSun, QinlinChoi, WookjinLiang, Chiu-kuoSun, ShaohuiChoi, A.Liang, FengSun, ShaohuiChon, KiLiang, HuaweiSun, ShuliChong, AlvinLiang, JinxingSun, WeiChong, Jo WoonLiang, Jun-RuiSun, XiangchengChong, PeterLiang, MengbingSun, YanhuaChoo, RaymondLiang, QiaokangSun, YeChou, HTLiang, Tsair-ChunSun, YunchuangChou, Jeffrey B.Liang, XishuangSun, YuruiChouvardas, SymeonLiang, XuefengSun, YuxiangChouvardas, Vasilios G.Liang, YanSun, ZhanliChow, Chi WaiLiao, BinSun, ZhenglongChow, JackyLiao, GuanglanSung, Tae HyunChowdhury, Ehsan H.Liao, GuanglanSur, RitobrataChowdhury, SazzadurLiao, Ming HanSurgenor, BrianChristian, John A.Liao, ShaolinSutherland, Garnette R.Christophe, BrunoLiao, XiaofengSutor, AlexanderChu, AlfredLiao, Yi-HungSutor, AlexanderChu, Cheng-ShaneLichti, DerekSuyama, RicardoChu, JinkuiLieberzeit, PeterSuzuki, KenichiroChu, Shu-ChuanLiebig, ThomasSuzuki, YoshioChu, Shu-ChuanLiebig, ThomasSvihlık, JanChu, Shu-ChuanLight, GlennSwager, TimChu, TianguangLikamwa, Patrick L.Swain, AkshyaChu, TianxingLim, Dong-WonSwami, NathanChu, VirginiaLim, Eng GeeSwartz, R. AndrewChu, Yen-hoLim, JaechanSynnes, KåreChuang, Cheng-hungLim, Khoon S.Sysoev, Victor V.Chuang, Cheng-LongLim, Loong-TakSzakmany, GergoChuang, Chun-Hsiang (Michael)Lim, SungjoonSzałachowski, PawełChuang, Kuo-ChihLim, Yah LengSzypłowska, Agnieszka Chuang, Po-Ya AbelLim, Yah LengSzantoi, ZoltanChuang, Yung-ChungLim, Yee YanSzeląg, WojciechChui, Hsiang-ChenLim, YujinSzewczyk, RomanChun, SejongLima, Fernando P.Sziebig, GaborChung, Jae-HyunLin, Bor-ShyhSzkaliczki, TiborChung, Tein-YawLin, ChiSzmacinski, HenrykChung, Tien-KanLin, Chien-YuSzöllősi, DanielChung, Wen-YawLin, Chih-MingTabbagh, AlainChung, YeonHoLin, Chih-mingTabib-Azar, MassoodChung, Young-seekLin, Chih-pingTachtatzis, ChristosChwa, DongkyoungLin, Chih-TingTae Hyun, KimCiabattoni, LucioLin, Chii-WannTahara, HironobuCianca, ErnestinaLin, Chin-FengTaheri, JavidCicchetti, RenatoLin, Chin-tengTajalli, ArminCicek, Paul-VaheLin, Chun-ChengTajbakhsh, Shahriar EtemadiCiecholewski, MarcinLin, Chung-WeiTakada, HideyukiCieslak, JérômeLin, FengTakada, SatoruCięszczyk, SławomirLin, Guo-ShiangTakahama, SatoshiCifuentes G., Carlos AndrésLin, Hsiung-ChengTakahashi, SatoruCigna, FrancescaLin, Hung-YinTakashima, KazutoCikajlo, ImreLin, Hung-yinTakei, KuniharuCimato, StelvioLin, Jaw-RenTaki, HirofumiCiminelli, CaterinaLin, King-Ip (David)Talei, AminCiminelli, CaterinaLin, Ming-BoTalens, ClaraCinar, EyupLin, Po-ChiangTallman, Tyler N.Cionca, VictorLin, QianqianTalvitie, JukkaCiosek, PatrycjaLin, SanbaoTamás, LeventeCipresso, PietroLin, Shih-ChunTamil, LakshmanCipriani, ErnestoLin, TaoTamura, HirokiCirani, SimoneLin, Tu-LiangTan, Chee-KeongCiriello, ValentinaLin, WeiTAN, Chuan Hao GrantCiuonzo, DomenicoLin, XiangguoTan, Do DuyCiuonzo, DomenicoLin, Yang-weiTan, Hwee-PinkClark, Adrian F.Lin, Yaw-jenTAN, JiajieClarke, KennethLin, Yen-HengTan, Kui-ThongClarke, Peter J.Lin, Yuan-PeiTan, Le ThanhClarke, Tomas G.Lin, Yuan-PinTan, LianshengClaudel, Christian G.Lin, Yuan-PinTan, QingClausen, IngelinLin, YucongTan, Say HwaClausmeyer, JanLinden, MariaTan, U-XuanCliffel, David E.Lindsey, Brooks DTan, WilliamCobo, Manuel JesusLing, Keck VoonTanaka, MasayukiCoburn, Craig A.Ling, Man HoTanaka, YoCocconcelli, MarcoLing, PeiTang, FeiCodetta-Raiteri, DanieleLing, SteveTang, FujianCoelho, João PintoLing, WingkuenTang, JianCogliati, SergioLing, ZhigangTang, LihuaColaizzi, PaulLingua, AndreaTang, LiqiongColao, FrancescoLinguet, LaurentTang, Lu-AnCole, MattLinker, RaphaelTang, ShijunCole, Matthew T.Lipka, TimoTang, XinhuaColeman, David J.Lipsett, MikeTang, YiCollins, ScottLissenden, CliffordTang, YueColtin, BrianLiu, AnfengTani, HirofumiColtro, Wendell Karlos TomazelliLiu, AnyiTanikawa, TomonoriCompagnone, DarioLiu, BifengTao, JunliangCompère, ChantalLiu, Bing-hongTao, LeiConci, NicolaLiu, ChengliangTaoukis, PetrosConforto, SilviaLiu, Chi (Harold)Tapu, RuxandraCong, XiaoyingLiu, ChuanjunTarchi, DanieleConnesson, NathanaelLiu, ChunhuaTardif, Pierre MartinCooke, MattLiu, ChunweiTarpanelli, AngelicaCoors, VolkerLiu, DapengTartagni, MarcoCorbellini, GiorgioLiu, DatongTasca, FedericoCorcoran, PeterLiu, GuigenTaseska, MajaCordero Fuertes, Juan AntonioLiu, GuochengTassou, Chrysoula C.Cordoba, AlbertoLiu, GuoliangTatlas, Nicolas-AlexanderCorigliano, AlbertoLiu, HaijunTaurino, IreneCorrea, Daniel S.Liu, HaitaoTavakkoli, AlirezaCorres, JesusLiu, HongTavío, María Del MarCortellessa, GabriellaLiu, HongboTay, Jian WeiCoskun, BulutLiu, HuafengTaylor, HaydenCosta, J MiguelLiu, JiangTeal, PaulCosta, MaiceLiu, JianxiTeather, Robert J.Costa, ManuelLiu, JifengTee, Benjamin Chee-KeongCosta, NunoLiu, JindongTeixeira, António J. S.Costa, RicardoLiu, JuTeixeira, Bruno O.s.Costa García, AgustínLiu, JuewenTeixeira, FernandoCostanzo, SandraLiu, JunTemko, AndriyCostello, Ben De LacyLiu, JunTen, Chee-WooiCottyn, JohannesLiu, KarenTendero, YohannCouceiro, RicardoLiu, LiangTeng, Wei-ChungCoutaz, JoelleLiu, LiangguiTenje, MariaCoviello, GiuseppeLIU, MingTennina, StefanoCovington, JamesLiu, MingTennina, StefanoCozzolino, AnthonyLiu, Name: JingweiTennina, StefanoCozzolino, DanielLiu, NanTeodoriu, CatalinCragg, PeterLiu, PeixiangTeodoro, A. C.Cragg, Peter J.Liu, QiongTerence, S. P. SeeCramer, MichaelLiu, QuanhuaTerroso, FernandoCrescentini, MarcoLiu, Shing-hongTesta, GenniCrespo, AlfonsLiu, ShufanTestoni, NicolaCrise, AlessandroLiu, ShutianTeti, RobertoCristiano, RobertoLiu, TaoTetienne, Jean-PhilippeCristina, Ana MajarenaLiu, TaoTeunissen, PeterCristina, Gaitan NicoletaLiu, TianshuTeunissen, Peter. J. G.Croft, Paul J.Liu, TingyiThaduri, AdithyaCruz, Homero ToralLiu, WeiTheilmann, FlorianCuadras, ÀngelLiu, WeitingTheodorakopoulos, IliasCubo, JavierLiu, WeiweiTheodoridis, EvangelosCuesta González, EduardoLiu, WenTheuwissen, AlbertCui, SongLiu, Wen-ChauThibault, PierreCui, Tie JunLiu, Wen-FungThielemann, ChristianeCui, XiaoweiLiu, WenzhongThielens, ArnoCui, YaoyaoLiu, XianweiThomas, FedericoCui, YiLiu, XiaoguangThomas, Jean BaptisteCui, ZiqiangLiu, XiaohanThomas, Jean-BaptisteCulshaw, BrianLiu, XinThomas, Stewart J.Culshaw, BrianLiu, XintaoThomason, WadeCulverhouse, PhilipLiu, XuThomaz, EdisonCumanan, KanapathippillaiLiu, XuanThompson, AlexCummins, GerardLiu, YahuiThompson, DavidCunha, MárioLiu, YangThompson, LeeCuriac, Daniel-IoanLiu, YangThompson, Mary KathrynCvejić, ŽeljkaLiu, YangThramboulidis, KleanthisCzechowski, NikodemLiu, Yao-HongTian, FengchunCzerwinski, DariuszLiu, YaoliTian, Gui YunD’Addona, Doriana M.Liu, YongbinTian, JieD’Alonzo, MarcoLiu, YonggangTian, LinDa Silva, Leandro DiasLiu, YunTian, XiaohuaDa Silvab, Joaquim C.g. EstevesLiu, Yung-tienTian, YanqingDabbicco, MaurizioLiu, YuxiangTian, YueDabbicco, MaurizioLiu, ZenghuaTian, ZhenhuaDabove, PaoloLiu, ZhengTiitta, MarkkuDae-Young, KimLiu, ZhengTimmermans, HarryDagdeviren, CananLiu, ZhidanTing, Ching-HuaDaggupati, PrasadLiu, ZhigangTiropanis, ThanassisDaghia, FedericaLiu, ZhiyuanTisan, AlinDahmen, Jeremy L.Liu, ZipingTischler, DirkDai, Henry Hong-NingLivache, ThierryTiu, Brylee David B.Dai, JishengLizcano, DavidTivive, Fok Hing ChiDai, LinglongLlagunes, Josep Maria FontTiwari, AshutoshDai, XuewuLlibre, Jean-FrançoisTiwari, RajeshDai, YuchaoLlinas, JamesTjin, Swee ChuanDamas, MiguelLlobet, EduardTkachenko, PavloD'Amato, FrancescoLlorens, JordiTkacz, EwarystD'Amico, ArnaldoLloréns, RobertoTobiszewski, MarekDamigos, MatthewLlorens Calveras, JordiToda, KeiDán, GyörgyLloret-Mauri, JaimeTodorovic, VladimirDanaher, TimLo, AnthonyTognetti, AlessandroDandekar, Kapil R.Lo, Chien-fongToibero, Juan MarcosD'andreagiovanni, FabioLo, Huang-MuTok, AndreDanes, PatrickLo, Shou-ChihTokumitsu, MasahiroDani, Keshav M.Lo, Yu-hwaTokunaga, EijiDaniela, CarrionLo, Yu-LungToledo-Moreo, RafaelDaniele, MichaelLo Bello, LuciaTollner, Ernest W.Daniele, PasseriLo Cigno, RenatoTomarchio, OrazioDantcheva, AntitzaLocatelli, ClinioTomassetti, MauroDante, AlexLockhart, ThurmonTomaszuk, DominikDao, Son V. T.loddo, donatoTomintz, MelanieDarehshoorzadeh, AmirLoessner, DanielaTomita, ShunsukeDargahi, TooskaLogsdon, Sally D.Tomlin, Nathan ADarmanin, ThierryLoh, KennethTommaselli, Antonio M. G.Das, Anshuman J.Lohan, Elena SimonaTomovsk, RadmilaDas, AruneemaLohstroh, AnnikaTompkin, JamesDas, JagotamoyLoizos, AndreasTong, WeitianDas, NarottamLomas-Vega, RafaelTöreyin, HakanDash, PrasanjitLong, Dan S.Torres, MagdaDaskalopoulos, IoannisLong, RunTorres, MiguelDasog, MitaLongo, DomenicoTorres, RominaDau, Van ThanhLongstaff, Andrew PeterTorres-Gomez, IsmaelDaum, WernerLongstaffe, James G.Torres-Sospedra, JoaquinDavalli, AngeloLopes, Ana C.Torricelli, DiegoDavari, HosseinLopez, Antonio ManuelTorsi, LuisaDavidsson, PaulLópez, Pedro PerisTortolini, CristinaDavies, ClaireLopez Dekker, PacoTorunbalci, Mustafa MertDavis, Tyler WLopez Riquelme, Juan AntonioToschi, IsabellaDavy, MatthieuLópez Villanueva, Juan AntonioToshiyoshi, HiroshiDawidowicz, AgnieszkaLopez-Amo, ManuelToth, GezaDay, Stephanie S.Lopez-Higuera, Jose MiguelTotis, G.De, SuparnaLópez-Lorente, Ángela InmaculadaTouhafi, AbdellahDe Adana, Francisco SaezLópez-Matencio, PabloTowfighian, ShahrzadDe Almeida, Hyggo OliveiraLopez-Molina, CarlosTran, Gia KhanhDe Araújo, Gustavo MedeirosLópez-Nicolás, GonzaloTran, Huy N.QDe Baere, IvesLopomo, NicolaTran, Le-NamDe Bei, RobertaLopresto, ValentinaTran, Ngoc-TrungDe Boer, GijsLosada-Perez, PatriciaTraver, VicenteDe Donno, DaniloLoscri, ValeriaTraversi, GianlucaDe Gracia, PabloLoseto, GiuseppeTravieso-Gonzalez, Carlos M.De Grande, RobsonLotfi, AhmadTrefzer, MartinDe Jesús Ku Herrera, JoséLoukas, GeorgeTrémeau, AlainDe La Cruz Llopis, Luis J.Louro, PaulaTrevathan, JarrodDe La Escalera, ArturoLoutas, TheodorosTrichard, FlorianDe La Escosura, AlfredoLovisolo, LisandroTripathy, MalayDe La Fuente, Jesús GrajalLozano, GabrielTriviño, AliciaDe La Fuente, RaúlLozano, Jesús FernándezTrögl, JosefDe La Oliva, AntonioLozano Rogado, JesusTroglia Gamba, MicaelaDe La Piedra, AntonioLu, Amy S.Troiani, EnricoDe La Torre, ÁngelLu, Bao-LiangTrommer, Gert F.De La Torre Díez, IsabelLu, Chia-JungTroncoso-Pastoriza, Juan RamónDe Marchi, LucaLu, Chun-ShienTrontelj, JanezDe Marcos, SusanaLu, GanhuaTrossen, DirkDe Matteis, MarcelloLu, GeyuTrovati, MarcelloDe Meo, PasqualeLu, Hai-hanTroynikov, OlgaDe Mesnard, LouisLu, HongguangTruong, Thuy T.De Sanctis, MauroLu, JianTruong-Hong, LinhDe Teixeira Da Encarnação, JoãoLu, MingquanTrusov, AlexanderDe Vargas Sansalvador, Isabel M. PerezLu, PingTsai, Du-MingDe Vito, SaverioLu, RongxingTsai, Kuo-YuDean, RobertLu, SiliangTsai, Meng-TsanDebliquy, MarcLu, TaoTsai, MichingDehghanian, VahidLu, WenlianTsai, Pei-YunDei, MicheleLu, XiaodongTsai, Wen-ChiDel Ama, Antonio J.Lu, YanTsai, Yi-Chang JamesDel Campo, JavierLu, YipengTsakalof, ADel Castillo Vazquez, MiguelLu, YueTsantili, EleniDel Valle, ManelLucas, GaryTseng, Kevin C.Delashmit, WalterLuches, ArmandoTseng, Yi-HsingDelepine-Lesoille, SylvieLucibello, AndreaTsigaridas, Georgios N.Dell’Isola, MarcoLuckner, MarcinTsiigkaridis, John (Ioannis)Della Corte, Francesco G.Łuczak, SergiuszTsioukas, VassiliosDell’Aglio, DanieleLudeno, GiovanniTsiourlis, GeorgiosDell’Olio, FrancescoLudwig, FrankTsipouras, MarkosDelnavaz, AidinLuengo García, DavidTsow, Francis (Tsing)Delrue, StevenLughofer, EdwinTsutsui, MinoruDel-Val-Noguera, ElenaLühmann, TessaTu, MichaelDemetriadis, StavrosLui, AntonyTuhkala, MarkoDemo Souza, RichardLui, King-shanTuma, JiriDemosthenous, AndreasLuigia, SabbatiniTung, Ryan C.Dempsey, Nora M.Luján, Jose Luis PozaTung Chong, David WongDen, WalterLukaski, HenryTuononen, Ari JuhaniDe Witte, Luc P.Luo, ChaominTura, AndreaDeng, RuilongLuo, Ching-HsingTurco, AntonioDeng, ShengyuanLuo, ChunboTurksoy, KamuranDeng, YanshaLuo, Jia-NingTurley, Glen A.Deng, ZhengmiaoLuo, JunTurner, ChrisDeng, ZhidongLuo, XichunTuset-Peiro, PereDeng, ZhihongLuo, YanhuaTuukkanen, SampoDenis, ProshlyakovLuo, YanjiaTwu, Ruey-ChingDenoual, MatthieuLupan, OlegTzallas, AlexandrosDeo, RandhirLupi, CyrilTzallas, AlexandrosDepari, AlessandroLutton, Rebecca E.M.Ubertini, FilippoDeptuch, Grzegrz W.Luzi, GuidoUbertini, FrancescoDeravi, Leila F.Lv, ShaolinUchikoba, FumioDer-Ming, MaLv, TiejunUddin, ZiaDesai, AlokLynch, Christopher S.Ueda, HiroshiDeschinkel, KarineM. Reindl, LeonhardUgaz, VictorDeshpande, KshitijaMa, Andy JinhuaUgazio, SabrinaDesmaris, VincentMa, ChangzhengUgbolue, Ukadike ChrisDeSouza, GuilhermeMa, Chien-chingUgulino, WallaceDestain, Marie-FranceMa, Dik-LungUhring, WilfriedDevendran, CitsabehsanMa, Guo-mingUmek, AntonDevkota, JagannathMa, Huiüney, MuratDeVries, J. HansMa, HuiUno, ShigeyasuDey, SatadruMa, JiajuUr Rehman, MasoodDezert, JeanMa, JianguoUrbanchek, Melanie G.Dezfouli, BehnamMa, JianguoUrbanová, PetraDhakal, SagarMa, JiemingUriarte, Rafael BrundoDhananjay, AdityaMa, LinUrquiza, LuisDhaou, RiadhMa, LonghuaUrueña, ManuelDhir, AmanMa, XiaoleiUrwyler, PrabithaDi, KaichangMa, YangjinUrzaiz, GabrielDi Barba, PaoloMa, Yuan-RonUsamentiaga, RubénDi Bisceglie, MaurizioMaaoui, ChoubeilaUslander, ThomasDi Capua, R.Macaire, LudovicUslar, MathiasDi Gennaro, S.Maccari, LeonardoUßmüller, ThomasDi Martino, GerardoMacDonald, BruceUttenthaler, StefanDi Natale, CorradoMacé, MarcUysal, IsmailDiamant, RoeeMach, PavelV Kennedy, JohnDianat, PouyaMachida, MasashiVagedes, JanDiao, XunxingMacias-Lopez, Elsa MaríaVaghefi, RezaDias, JorgeMaciejewska, DorotaVaglio Laurin, GaiaDias, Luís G.MacKinnon, DavidVainio, MarkkuDias, M. BernardineMacnae, James C.Val, ThierryDias, PauloMadadi, HojjatValencia, José F.Díaz-Báñez, José MiguelMadasamy, ThangamuthuValentini, PPDíaz-Cruz, José ManuelMadhu, NileshVales Alonso, JavierDickert, FranzMaeda, IsamuValkama, PekkaDieber, BernhardMaeda, NobutakaVallati, CarloDiethe, TomMaes, WillyValli, EnricoDietzel, StefanMaestre, RafaelValova, IrenDijksman, J. FritsMaffeis, ThierryVan Bennekom, W.P.Dikaios, NikolaosMaffiuletti, Nicola A.Van Blerkom, DanielDilo, ArtaMafra, SamuelVan De Moortele, Pierre-FrançoisDilo, ArtaMagazzeni, DanieleVan De Wetering, FerdiDilonardo, ElenaMagee, JohnVan Den Abeele, FlorisDincer, CanMager, DarioVan Den Dobbelsteen, J. J.Ding, DaweiMaglaras, LeandrosVan Den IJssel, JoseDing, GuoruMaglaras, LeandrosVan Den Noort, Josien C.Ding, GuoruMagno, MicheleVan Der Meer, JohanDing, HuiMagno, MicheleVan Der Spiegel, JanDing, Jow-LianMagnusson, MartinVan Der Zande, DimitryDing, PingpingMagnusson, RobertVan Dinh, AnhDing, XiaoyunMahalle, Parikshit N.Van Hirtum, AnnemieDing, YongMaheshwari, VivekVan Kleunen, WouterDING, ZhizhongMahfuzul Islam, A.K.M.Van Roy, PeterDinh, Thien X.Mahgoub, MohamedVan Walree, PaulDinham, MitchellMahmoud, Mohamed M. E. A.Vandelanotte, CorneelDionysis, BochtisMaillé, PatrickVandenbroucke, JustinDios, J.r. Martinez-deMailly, F.Vangelista, LorenzoDipietro, LauraMaimour, MoufidaVanhoestenberghe, AnneDiraco, GiovanniMak, Peng UnVanli, O.ArdaDixon, CoryMakarov, VAVardasca, RicardoDlott, Dana D.Makhoul, AbdallahVardoulis, OrestisDo, Frederic C.Malakar, Nabin K.Varga, MariánDobbins, ChelseaMalakooti, Mohammad HVargas-Rodriguez, EverardoDobre, CiprianMalaney, RobertVasconcelos, FranciscoDomaneschi, MarcoMalara, PietroVashist, Sandeep KumarDomenico, GrimaldiMaldague, XavierVasic, MilosDominguez Brito, Antonio CarlosMaleki, SinaVasilakos, Athanasios V.Donaldson, NickMalinge, Jan-MarcVasiljević-Radović, DanaDonarelli, MaurizioMallios, AngelosVasseur, PascalDonato, Loreto DiMalumbres, Manuel PerezVatavu, AndreiDonelli, MassimoMalvezzi, MonicaVÁZQUEZ CORRAL, JAVIERDong, BoMamalis, BasilisVazquez Garcia, CarmenDong, DananMamun, QuaziVecchio, AlessioDong, FengzhongMancini, AdrianoVega Pérez, GraciaDong, HaifengMandapati, Ramakrishnam RajuVehkaoja, AnttiDong, HaiweiMandow, AnthonyVelazquez, RamiroDong, JunhangManduchi, RobertoVelázquez, RamiroDong, LeiManfrinato, VitorVelotta, RaffaeleDong, LiliMangan, MichaelVeltink, Peter H.Dong, WeiMani, VeerappanVenditti, IoleDong, XiaochenManicardi, AlexVeneri, OttorinoDong, XichaoManickam, PandiarajVenkatasubramanian, KrishnaDong, ZiqianManikas, AthanassiosVenkatesan, RamachandranDonha, Décio C.Manini, Todd MVentura, Joao OliveiraDonis-Gonzalez, Irwin R.Mankin, R. W.Venus, JoachimDonoso, YezidMankodiya, KunalVerd, JaumeD’Orazio, TizianaMann, Danny D.Verdugo, Antonio SoriaDorronzoro, EnriqueManneback, PierreVergara-Laurens, Idalides J.Doryab, AfsanehMännel, BenjaminVerhagen, Sandrados Santos, Alcindo A.Manninen, AlbertVerhoeven, BenDos Santos, DanielMannini, AndreaVerikoukis, ChristosDos Santos, M. SergeManogharan, Guha PrasannaVermeersch, PatriciaDosen, StrahinjaManolis, CharlieVerschae, RodrigoDossi, NicolòManorathna, PrasadVetrella, Amedeo RodiDou, WanchunMäntele, WernerVettore, AntonioDoulamis, AnastasiosMantri, DnyaneshwarVezzetti, EnricoDoulamis, Nikolaos D.Manuri, FedericoViani, FedericoDouligeris, ChristosManzanera, AntoineViani, FedericoDoumenis, GregoryManzino, Ambrogio M.Vicentini, FedericoDoweck, IlanaMao, HongjuVictores, Juan G.Down, MichaelMao, XiangleiVidal-Verdú, FernandoDowrick, ThomasMao, XinhuaVidoni, RenatoDrager, KathyMarani, RobertoViegas, Diana CatarinoDragone, MauroMarc, LesturgieVieira, Douglas Alexandre GomesDragut, LucianMarcello, JavierVieira, ManuelaDrap, PierreMarcelloni, FrancescoVien, Quoc-TuanDrews, PauloMarchiano, RégisVignoli, ValerioDreżewski, RafałMarco, BietresatoVignudelli, StefanoDrobizhev, MikhailMARCO, CARMINATIViik, JariDu, DongMarcolin, FedericaVila, Xosé A.Du, ShengMarcos, MarcosVilanova, XavierDu Toit, HendrikMargara, AlessandroVilela, D.Duan, ZhanshengMargaria, DavideVilella, EvaDuann, Jeng-Ren María Enguita, JoséVilla, FedericaDubey, AshishMariani, AndreaVilla, PaoloDubuc, DavidMariani, StefanoVilladangos, JesusDubuc, DavidMarignetti, FabrizioVillagra, FedericoDuc, G.Marín, JavierVillani, Maria GabriellaDuchaine, VincentMarinesco, StéphaneVillano, MichelangeloDucournau, GuillaumeMarino, AlessandroVillegas, DolorsDudczyk, JanuszMarino, ArmandoVilquin, BertrandDudley, SandraMarinoni, MauroVincenzo, MinutoloDuerkop, AxelMariotto, IsabellaVines, PeterDuggen, LarsMarita, TiberiuViola, Ignazio MariaDuhovic, MiroMarkham, AndrewViollet, StéphaneDumee, LudovicMarkopoulos, PanosVirén, TuomasDunai, LarisaMarks, MichałVisser, FleurDunbar, AlanMaroufi, MohammadVisy, CsabaDunbar, Alan D.F.Marques, Manuel Joaquim BastosVítek, StanislavDuncan, Alec JMarques, Manuel JorgeVitetta, Giorgio MatteoDuong, Quang TrungMarrone, StefanoVitucci, Enrico MariaDuquennoy, SimonMARS, KamelVivas Albán, Oscar AndrésDuraia, El-shazly M.Marsh, LiamVivet, DamienDuro, NatividadMartalo, MarcoVlacheas, PanagiotisDuro, RichardMartalò, MarcoVlahogianni, EleniDutton, NealeMartalò, MarcoVo, Ba-NguDuveiller, GregoryMartin, DavidVogel, ThomasDworak, VolkerMartin, SergioVoglhuber-Brunnmaier, ThomasDyer, AdrianMartín, DavidVoiculescu, IoanaDyo, VladimirMartín, FerranVoigtländer, BertDzamba, TomMartinez, EdgarVoitechovic, EditaDzantiev, BorisMartinez, Jose FernanVolanti, Diogo PaschoaliniDziuda, ŁukaszMartinez, Jose MariaVolos, ChristosDziurdzia, PiotrMartinez, Ramses V.Volosencu, ConstantinEastwood, PeterMartínez, José F.Von Frese, RalphEckert, MartinaMartinez de Dios, José RamiroVon Jan, UteEdirisinghe, MohanMartínez Martín, DavidVosch, TomEdiriweera, SisiraMartinez-Gomez, Norma CeciliaVu, Ngoc SonEdwan, EzzaldeenMartínez-Martí, FernandoVulfson, LeonidEdwan, EzzaldeenMartini, AlbertoVulpe, AlexandruEdwards, R.S.Martini, RobertoVuolo, FrancescoEftekhar Azam, SaeedMartin-Lopez, SoniaVu-Van, HiepEftekhar Azam, SaeedMartino, LucaVyskočil, VlastimilEgami, YasuhiroMartins, Wallace AlvesWaclaw, BartekEgea-Roca, DanielMartins-Filho, Luiz S.Wada, DaichiEgger, JanMartí-Vargas, J.R.Wada, YujiEhlen, J. ChristopherMartowicz, AdamWade, Eric R.Eich, MarkusMartyniuk, MariuszWagner, BernardoEiza, Mahmoud HashemMaruyama, SatoshiWagner, PatrickEiza, Mahmoud HashemMarxer, RicardWagner, Stefan RahrEizenberg, HananMarzani, AlessandroWagner, StephanEizenman, MosheMasayuki, AnyojiWagner, ThorstenElberink, Sander OudeMascarenas, DavidWahaia, FaustinoEl-Darymli, KhalidMaschmann, MatthewWajman, REl-Darymli, KhalidMasek, VlastimilWalczak, KrzysztofEldessouki, MohamedMasiero, AndreaWalkowiak, KrzysztofEliasson, JensMasini, Barbara MaviWallhoff, FrankEllis, TimMaslowski, AndrzejWalter, Johanna GabrielaElm, Matthias T.Mason, AlexWalter, Jonathan PElmenreich, WilfriedMasone, CarloWalton, GabrielEl-Osery, Aly I.Masoodian, SalehWaltrich, GierriEl-Sheimy, NaserMasotti, DiegoWan, DuElsts, AtisMasouros, ChristosWan, FengElvira, VíctorMasseroni, DanieleWan, G.Y.Emadi, ArezooMassoth, MichaelWan, JiafuEmadi, ArezooMatagne, ErnestWan, JiafuEmardson, RagneMata-Moya, DavidWan, Kai TaiEmmi, LuisMataric, Maja J.Wan, ZhanmingEmokpae, LloydMatatagui, DanielWanderer, JonathanEndo, TatsuroMatekovits, LadislauWang, AiminEngelhard, NikolasMatellán Olivera, VicenteWang, AiningEnglebienne, GwennMatese, AlessandroWang, BangEnikov, Eniko T.Matko, VojkoWang, BinbinEnright, JohnMatlis, Eric H.Wang, CaixiaEnz, ChristianMatos, AníbalWang, ChaohuiEriksen, René LyngeMatson, Eric T.Wang, ChaonanErives, HectorMatsuda, NathanWang, ChengErkin, ZekeriyaMatsui, TakemiWang, Cheng-LiangErlebach, ThomasMatsuki, YohWang, Chi-KueiErnst, JosephMatsumura, KentaWang, Chiu-YenErro Betrán, María JoséMatsushita, KojiroWang, CongEscamilla-Ambrosio, Ponciano JorgeMatthäus, ChristianWang, CongEscareno, Juan-AntonioMatthews, PeterWang, DaiEscribano, Javier GómezMatthias, SchickWang, David ZhiweiEscudero, Alfonso PérezMaudes, JesusWang, DiEsfandiari, LeylaMaugey, ThomasWang, DongEsfandyarpour, RahimMaurits, Natasha M.Wang, Dung-AnEshagh, MehdiMavris, DimitriWang, EricEspensen, EricMay, StefanWang, Faming (Felix)Espinosa, FelipeMaybank, SteveWang, FeiEspinosa-Urgel, ManuelMayer, DirkWang, Gou-JenEsposito, AntonioMazilu, SinzianaWang, GuijinEsposito, ChristianMazurek, PrzemysławWang, GuodongEstatico, ClaudioMazza, ClaudiaWang, HaiEsterle, LukasMazzaro, GregoryWang, HaidongEsteve-Romero, JosepMazzei, DanieleWang, HeEstevez-Tapiador, Juan M.Mazzeo, Pier LuigiWang, HongboEstima, Jorge OliveiraMazzon, RiccardoWang, HuaxiangEstrella, Júlio CezarMazzoni, AugustoWang, Hui-MingEugenio, FranciscoMcCarthy, UltanWang, Hung-YuEvangelatos, OrestisMcCullagh, PaulWang, JiachouEvangeli, CharalambosMcDougall, KevinWang, JianEvtugyn, GennadyMcEwan, AlistairWang, JiangzhouExadaktylos, AristomenisMcFarlane, NicoleWang, JianpingFabian, MattiasMcGibney, AlanWang, JieFabien, FerreroMcGough, Robert J.Wang, JigangFabregas, ErnestoMcGrath, DanielWang, JingchuanFacchi, ChristianMcGrath, RobertWang, JinzhiFacchinetti, AndreaMcKeague, MaureenWang, JuFacchinetti, TullioMcKeever, SusanWang, JunFaezipour, MiadMcKendry, JonathanWang, JunyuFaia, Pedro Manuel Gens Azevedo MatosMcKinley, BrettWang, KaiFaion, FlorianMcLamore, EricWang, Ke-ShengFalahi, GhazalMcNally, AmyWang, Kevin I.-K.Falciola, LuigiMcWilliam, StewartWang, LeiFalco, GianlucaMecella, MassimoWang, LeiFalco, GianlucaMedagoda, LashikaWang, Li (Lily)Falcone, FranciscoMedola, FaustoWang, LiangFalletti, EmanuelaMedrano, NicolásWang, LiangminFamaey, JeroenMedved, VladimirWang, LihuaFan, ChaoMedvidović-Kosanović, MartinaWang, LiuchengFan, JingMeek, Sanford G.Wang, LizheFan, Kuang-ChaoMehrabi, NaserWang, MiFan, PingyiMEI, LEIWang, Mu-chunFan, ZhiyongMeikle, William GWang, NingFanelli, FrancescoMejia-Beltran, EfrainWang, PengfeiFang, Da-GangMekhalfi, Mohamed LamineWang, PengtaoFang, FeiMelchiorri, ClaudioWang, PingFang, PengMeléndez, JoaquimWang, QingQingFang, RongxinMelendez-Pastor, IgnacioWang, QingyangFang, Shih-HauMeli, EnricoWang, QunmingFang, ShuhuaMelillo, PaoloWang, QunmingFang, WeiweiMellbye, Brett L.Wang, SenFang, X.Melo-Pinto, PedroWang, ShengkaiFanti, GiuliaMemedi, MevludinWang, Sheng-ShihFantuzzi, NicholasMen, YongjunWang, Sheng-ShihFarag, KarimMendez Zorrilla, AmaiaWang, TanyuFaria, Diego R.Mendizabal, JaizkiWang, TengFarias, GonzaloMendonca, AgnesWang, Tsan-pinFarid, Amro M.Menendez, HectorWang, WeisongFarinella, Giovanni M.Menezes, PauloWang, WeixingFarinelli, DanielaMeng, WenchaoWang, Wendy HuiFarr, ThomasMeng, XiangyuWang, Wenweifarris, StefanoMeng, ZhaozongWang, XiaFathy, AlyMenna, FabioWang, XiangrongFattahi, HereshMeola, CarosenaWang, XiangrongFavero, GabrieleMera, DavidWang, XiaofeiFay, Patrick J.Mercader, R.j.Wang, XiaohanFazio, PeppinoMercado, Karla P.Wang, XiaolingFekete, ZoltánMercorelli, PaoloWang, XinyuanFeldman, YuriMerryman Boncori, John PeterWang, XiuminFelici-Castell, SantiagoMertes, Carly M.Wang, XuguangFelipe, Cunha DomingosMesas-Carrascosa, Francisco JavierWang, YafeiFelis, IvanMesas-Carrascosa, Francisco-JavierWang, YanFelisberto, PauloMeseguer, RocWang, YanchengFelix, Anderson AndréMesquita, DanielaWang, YanzhiFeller, Jean-FrancoisMessina, MarcoWang, YaojinFelski, AndrzejMessori, LuigiWang, Yao-tienFemminella, MauroMestre, DanielWang, YiFeng, DongmingMetsis, VangelisWang, YiFeng, HuiMeulenberg, ElineWang, YideFeng, LianMeybodi, Morteza KhaleghiWang, YingFeng, QiboMiah, Abdul HalimWang, YiruFeng, ShaojunMiao, JianminWang, YishiFeng, TaoMichael, AronWang, YongxiangFeng, YongjiuMichael, PechtWang, YuFeng, Zhi HuaMichalas, AntonisWang, YuFentanes, Jaime PulidoMichalet, XavierWang, Yu (Winston)Fenzl, ChristophMichalos, GeorgeWang, YuboFerhan, Abdul RahimMichalska, AgataWang, YucaiFernandes, Emanuel M.Michalski, AndrzejWang, Yu-JenFernandes, Ricardo JorgeMicheli, AlessioWang, YulingFernandez, RoemiMicheli, DavideWang, YunFernández, EduardoMicheli, LauraWang, Yu-TeFernández Caballero, AntonioMichelson, DavidWang, ZengcaiFernández Melián, SuselMichihata, MasakiWang, ZhaohuiFernández Ramos, María DoloresMiddleton, Stuart E.Wang, ZhenxinFernández Romero, Juan ManuelMiettinen, JariWang, ZhiboFernández-Avilés, GemaMiguel, Gonzalo DeWang, Zizhong JohnFernandez-Llatas, CarlosMiguel, Juan C. SanWang, HaoFernández-Sánchez, CésarMiguel-Bilbao, Silvia DeWanninger, LambertFernando, CharithMihajlović, VojkanWard, Jamie A.Fernando, XavierMihaylova, LyudmilaWard, JonathanFerrari, GiorgioMikhaylov, KonstantinWard, KorenFerré-Borrull, JosepMikhelson, Ilya V.Ward, PhilFerrein, AlexanderMikkelsen, Lars PilgaardWargocki, PawelFerreira, Paula MaríaMikš, AntonínWarrensmith, StephenFerreira Da Silva, RafaelMilanés, VicenteWarren-Smith, StephenFerretti, GianniMilani, RobertoWarriner, KeithFerri, GiuseppeMilani, SimoneWasa, YasuakiFerrigno, LuigiMilazzo, AlbertoWashington II, Aaron L.Ferro, YannisMilazzo, CristinaWatanabe, TetsuyouFescenko, IljaMilella, AnnalisaWatanabe, YoshihiroFicco, MassimoMilic, DamjanWATEL, DimitriFidan, BarisMilillo, PietroWatier, BrunoFidanova, StefkaMilitino, Ana FernándezWatkins, A. NealFiedler, GoeranMiller, BorisWatson, Dennis G.Fiedler, MarkusMiller, JimmieWatson, RobertFields, TravisMilosevic, BojanWatson, Simon A.Figueiras, EditeMin, Hoon-KiWebb, Jacqueline F.Figueiredo, LinoMin, SedongWeber, AndreasFigurski, MariuszMin Aung, MyoWeber, Griffin M.Filip, AlexandraMinami, TsuyoshiWeber, JostFilleter, TobinMinamikawa, TakeoWebster, JohnFink, AndreasMinardo, AldoWegner, Karl DavidFink, Glenn A.Minas, GracaWei, FengFink, OlgaMinazzi, RobertaWei, HongjiangFinkel, PeterMinet, PascaleWei, Hung-YuFinocchiaro, PaoloMinussi, Carlos RobertoWei, LeiFioranelli, FrancescoMirabella, SalvoWei, PingFiorini, LauraMiranda, AdelaideWei, ShuangqingFischer, AndreasMirasoli, MaraWei, XiongFischer, AndrewMiribel, Pedro LuisWei, ZhenZhongFischer, GeorgMiron, SebastianWeigel, RobertFisher, Daniel K.Miroshnichenk, Andrey E.Weihnacht, ManfredFlechsig, Gerd-UweMirov, SergeyWeinberg, MarcFleming, AaronMirsky, Vladimir M.Weller, MichaelFletcher, CameronMirsky, Vladimir M.Wells, B. EarlFletcher, Steven J.Mirza, DibaWells, Brian JFleury, AnthonyMirza, DibaWells, IanFlowers, GeorgeMisgeld, BernoWen, Chih-YuFoerster, TheodorMisiakos, KonstantinosWen, ShengFoi, AlessandroMiska, MarcWencel, DorotaFonollosa, JordiMisra, DurgaWerneck, Marcelo MartinsFonollosa Magrinyà, JordiMitchell, JohnWerner, MartinFontaine, Jean-françoisMitchell, JohnWerthschützky, RolandFontana, MarcoMitloehner, Frank M.Wessels, BruceFontecave, JulieMitobe, KazuhisaWestafer, Ryan S.Fontecha, JesúsMitra, SrinjoyWestfeld, PatrickForbes, Alistair B.Mitra, SunandaWeyn, MaartenForczmanski, PawelMittendorfer, PhilippWhalley, JacquelineForeman, MatthewMiwa, TomioWheeler, DavidForghani, AlanMiyazaki, HiroyukiWhite, Daniel J.Forke, RomanMiyoshi, DaisukeWhite, DevinForlenza, GregoryMizsei, JánosWhite, Ralph E.Forner-Cordero, ArturoMizukaki, ToshiharuWhitehead, AnthonyForste, ChristophMizuno, HideakiWhitehead, Debra E.Forsyth, JasonMlynarczyk, JanuszWhitty, Mark AlbertFort, AdaMłyniec, AWiberg, MikaelFortino, GiancarloMo, HainingWicks, MichaelFortuna, LuigiModelski, JozefWickstrom, NicholasFortuna, LuigiMoeslund, ThomasWierzba, PawełFoschini, LucaMoh, SangmanWijnhoven, RobFosson, SophieMohd Yasin, FaisalWijshoff, RalphFossum, EricMohrehkesh, ShahramWiklundh, Kia C.Foster, ChristopherMohri, KaneoWildmann, NormanFoster, ChristopherMöhwald, HelmuthWilhelm, JayFoster, JamesMokhtari, SoroushWilkinson, BenFoti, DoraMölders, NicoleWilkinson, MaxwellFotiadis, DimitriosMolina-Markham, AndrésWiller, UlrikeFotiadis, DimitriosMöller, ReinhardWillers, JeffreyFotouhi, HosseinMolnar, GaborWilliams, Paul JFourati, NajlaMon, Yi-JenWilliams, TrishFowler, BoydMondal, TKWilliamson, GrantFraga, Mariana AmorimMongelli, Domenico Walter EdvigeWilson, Alphus D.Fragkiadakis, AlexandrosMongiardo, MauroWilson, BenjaminFrancesco, AlettaMonico, João Francisco GaleraWilson, CherylFrancis, DanielMonifi, FarazWilson, CyrilFrancois, AlexandreMonkawa, AkiraWilson, PeterFranke, DanielaMonno, YusukeWilson, William C.Franke, UweMonorchio, AgostinoWinterhalter, MathiasFrasca, MattiaMontanaro, MatthewWirtz, HannoFrascella, FrancescaMonteil, ThierryWiśniowski, PiotrFratini, AntonioMonteiro, Miguel P.Witlox, FrankFratoddi, IlariaMonteriù, AndreaWitrisal, KlausFrazier, PaulMontero, Rocío SánchezWoellenstein, JuergenFrazzon, Enzo MorosiniMontes, MarcosWójcikowski, MarekFreddi, AlessandroMontillet, Jean-philippeWojtas, JacekFrederic, EghiaianMontoliu Colas, RaulWolek, ArturFreitas, GustavoMontoya, AngelWon, MyounggyuFrench, AndrewMontoya, ÁngelWong, Angus K. Y.French, ToddMontoye, AlexWong, Danny K. Y.Frese, ChristianMontoye, AlexanderWong, JeniseFrese, RaoulMontoye, AlexanderWong, Kainam ThomasFreymueller, JeffMonzón, Mario D.Wong, Keith Man-ChungFreymueller, JeffMonzón-Hernández, DavidWong, Kin-hongFrick, VincentMoon, Hong ChulWong, Kok-SengFriedli, MichaelMoon, TaesupWong, Kwan KitFriedt, Jean-MichelMoon, WonkyuWong, LawrenceFriedt, Jean-MichelMooney, PeterWong, Lu ShinFriedt, Jean-MichelMoore, PhilipWong, Man-kinFrischholz, RobertMora, AndreWoo, Jong-KwanFrizera, AnselmoMora Jimenez, InmaculadaWook Baik, SungFrizera Neto, AnselmoMorabito, Francesco CarloWozniak, AdamFrodella, WilliamMorago, BrittanyWu, BaishengFroese, Robert E.Morais Dos Santos, RaulWu, BianFröhlich, Antônio AugustoMorales, SophieWu, CelimugeFrueh, CarolinMorales-Narváez, EdenWu, Chao-ChengFryskowska, AnnaMorales-Narváez, EdenWu, Chao-chengFu, ChanghongMorallon, EmiliaWu, Chung-Tse MichaelFu, DongfeiMorbidi, FabioWu, DiFu, WeihongMoreau, JulienWu, HangbinFuchs-Kittowski, FrankMoreira, AdrianoWu, Hau-tiengFuentes, SigfredoMorell, AntoniWu, HuamingFuhrmann, ThomasMoreno, FélixWu, HuananFujioka, KoukiMoreno, LuisWu, JiahuiFujiwara, EricMoreno, Miguel AngelWu, JianfengFukatsu, TokihiroMoreno Arboleda, Francisco JavierWu, JianjunFuller, Amelia A.Moreno-Munoz, AntonioWu, JianminFung, Wai-keungMoreno-Salinas, DavidWu, JiashengFurferi, RoccoMoreno-Valenzuela, JavierWu, JiyanFurusawa, HiroyukiMoreu, FernandoWu, Jui-yuFusiello, AndreaMorgan, SteveWu, KaishunFutagawa, MasatoMorgül, Ender FarukWu, LanG. Costa, Mário MMorillo, CarlosWu, LinGaboury, SebastienMORIMOTO, MasakazuWu, MingquanGabriel, EdgarMorioka, KazuyukiWu, MingquanGabrielli, AlessandroMORISAWA, YusukeWu, Ta YeongGachet Páez, DiegoMoron, CarlosWu, TaoGade, RikkeMorón, M. J.Wu, Tian-YauGagne, David JohnMorosi, SimoneWu, Wei-chiangGajda, JanuszMorozov, MaximWu, Wen-JongGala, Tekleab S.Morrey, DeniseWu, WentaoGaliana, IgnacioMorris, BrendanWu, XiaofengGaliano, Antonio BernáMorris, DanWu, XiaopingGallagher, Justin FrancisMorris, KcWu, Xiaoping (Frank)Galland, StéphaneMorris, Patricia A.Wu, XinkaiGalletti, ChristopherMorshed, BashirWu, YanGalli, RobertaMortazavi, Jack BobakWu, Yik-ChungGallo, DanieleMoscato, VincenzoWu, YuanmingGallo, PierluigiMoschitta, AntonioWu, YuleiGalluzzi, ValerieMoschou, Despina C.Wu, ZhengGalopin, NicolasMoshou, DimitriosWu, ZhishengGalstyan, VardanMoshou, DimitriosWujcik, EvanGalvan-Tejada, Carlos EricMotagh, MahdiWunderlich, Ch.Gambi, EnnioMotai, YuichiWürtz, Rolf P.Gambus, PedroMotta, GianmarioWysocki, LauraGamo, JavierMotte, ErwanWysocki, MarianGams, MatjažMounaix, PatrickXafenias, NikolaosGamse, SonjaMoura, JoseXia, ChunleiGan, NingMourtzis, DimitrisXia, LinyuanGan, Tat-HeanMousavi Lajimi, S. AmirXian, XiaojunGanchev, IvanMu, MingquanXian, YuezhongGandino, FilippoMueller, Jennifer L.Xiang, MingmingGanzha, MariaMuhammad, BilalXiang, TaoGao, BinMujica, Luis EduardoXiang, WeiGao, David (Zhiwei)Mukheibir, PierreXiang, XianboGao, DeyunMukherjee, Partha SarathiXiang, ZhiyuGao, HuiMuleo, RosarioXiao, BaihuaGao, HuijunMuller, AlexandruXiao, JizhongGao, JingxiangMuller, CobusXiao, LiGao, Li QianMunafo, AndreaXiao, LiangliangGao, ShenghuaMunir, Kashif M.Xiao, QingjunGao, YandongMunir, TajXIAO, WenGao, YueMuñoz, JoseXiao, YangGao, ZhanMuñoz-Ferreras, José-MaríaXiao, YuGao, ZhiqiangMuñoz-Gea, Juan PedroXiao, YunfengGarcia, JordiMunz, MichaelXiao, ZhuGarcia, JorgeMura, AndreaXie, GuangzhongGarcia, Miguel AngelMurakoshi, HidejiXie, HuikaiGarcia, RafaelMuralikrishnan, BalaXie, JackyGarcía, AntonioMurao, KazuyaXie, JinGarcía, FernandoMurayama, RiichiXie, LeiGarcía, Juan C.Muroyama, MasanoriXie, WenjingGarcía Vázquez, Juan PabloMuscato, GiovanniXie, XinGarcia Zapirain, BegoñaMusić, JosipXie, ZhijianGarcía-Alonso, AlejandroMusolino, GiuseppeXin, XiaopingGarciaCarrillo, Luis RodolfoMussetta, MarcoXing, FeiGarcía-Casado, JavierMyers, MatthewXing, XiaofeiGarcía-Chamizo, Juan ManuelMyers, MikeXiong, JieGarcia-Fidalgo, EmilioMysore Harish, KiranXiong, JipingGarcía-Lamont, FaridMyung, HyunXiong, KaiqiGarcía-Miguel, Javier PortelaNabki, FrédéricXiong, XingguoGarcia-Morchon, OscarNag, SreejaXu, ChenrenGarcía-Orellana, Carlos-javierNagai, HirokiXu, H.LGarcia-Perez, ArturoNaganawa, ShigemiXu, JiaGarcia-Sanchez, FelipeNagarajan, SudhagarXu, JiaGargiulo, GaetanoNaghdy, FazelXu, JiangtaoGargiulo, Gaetano DNagl, StefanXu, JiawenGarmatyuk, DmitriyNagy, Peter B.Xu, JieGarrido, AngelNaik, Ganesh R.Xu, Li DaGarrido, PiedadNaït-Abdesselam, FaridXu, LinlinGartia, Manas RanjanNakahata, KazuyukiXu, MaotianGasbarri, PaoloNakajima, HironoriXu, RichardGashinova, MarinaNakajima, ShinichiXu, RuiGąska, AdamNakamori, SeiichiXu, TingtingGaspera, Enrico DellaNakamura, TomoyaXu, WeiheGates, ByronNakano, MichihikoXu, XiGatesman, Andrew J.Nakatani, TomoyaXu, XiaosuGatto, EmanuelaNakayama, MasaharuXu, XinGaudioso, ManlioNakhmani, ArieXu, YiGaudou, BenoitNakonieczna, AnnaXu, YongGaugel, TristanNam, YunjunXu, YongjunGaur, PramodNam, YunyoungXu, YougenGautier, MatthieuNanayakkara, ThrishanthaXu, YubinGavrilova, Marina L.Naqvi, MohsenXu, YuquanGazzola, EnricoNaqvi, Syed MohsenXue, BindangGe, CuiNarakathu, Binu BabyXue, KaipingGe, MaorongNaranjo, Fernando B.Xue, WeiGe, MaorongNaranjo, José EugenioXue, XinyuGE, XiaoliangNaranjo-Hernández, DavidYahiaoui, Riad.Ge, XudongNaranjo-Rodriguez, IgnacioYamada, TohruGebbers, RobinNarayanan, Ram M.Yamagishi, MasaoGeddis, Demetris L.Nascimento, Erickson R.Yamamoto, ToruGeem, Zong WooNaseer, NomanYamane, DaisukeGeissler, DanielNasipuri, AsisYamazaki, ToshimasaGelenbe, ErolNassar, SamehYamazato, TakayaGemeiner, PeterNassif, HaniYamazoe, HirotakeGendron, PaulNatraj, VijayYan, DongGeneiatakis, DimitrisNatschläger, ThomasYan, FengGeng, GuangchaoNatsuaki, RyoYan, Jie-BangGeng, JianghuiNavaei, MiladYan, MingGeng, JianghuiNavarro, Antoni PérezYan, MingyuanGennarelli, GianlucaNavarro, Javier LorenzoYAN, RUI-JUNGenovese, AlessandroNavarro, JMYan, RuqiangGentilini, IacopoNavarro, VictorYan, Wai YeungGeorgiadis, CharalambosNavarro-Pedreño, JoseYan, Xin-QingGeorgiadis, CharalamposNavarro-Serment, Luis ErnestoYan, ZhijunGeorgios, KambourakisNavas González, RafaelYang, BoGeorgoulas, ChristosNavas-Cortés, Juan A.Yang, BoGeorgoulas, GeorgeNeasham, JeffreyYang, CeGeorgoulas, GeorgeNecsoiu, MariusYang, ChangxiGeorgoulias, AristeidisNeedoba, JosephYang, ChengGerber, DoronNegi, SandeepYang, Chuan-KaiGerling, KathrinNehorai, AryeYang, ChungangGeršak, GregorNeitzel, FrankYang, DongminGestal, MarcosNekongo, Emmanuel EYang, Eileen Chih-YingGetzin, StephanNemova, GalinaYang, GengGhafar-Zadeh, EbrahimNepa, PaoloYang, Hee-DeokGhanbarpour, HabibNeska, AnneYang, Hung-WeiGhannad-Rezaie, MostafaNesterova, Irina V.Yang, JiachenGharechelou, SaeidNeumann, Patrick P.Yang, JinfengGhayesh, Mergen H.Nevat, IdoYang, Jinn-MinGheorghiu, Razvan AndreiNewe, ThomasYang, Laurence TGherlone, MarcoNewman, GregoryYang, LiGhiani, GiuseppeNezhad, Amir SanatiYang, LianxiangGhiass, Reza ShojaNg, AndyYang, MaoGhica, MarianaNg, JasonYang, MingGhidoni, StefanoNg, Wai TungYang, Ming-DerGhione, GiovanniNgan, HenryYang, PengGhisi, AldoNgo, Ha-duongYang, PoGhodssi, RezaNguyen, Duy H.Yang, QingGhoreishizadeh, Sara S.Nguyen, HoangYang, Seong WookGhosh, AyanjeetNguyen, Hung K.Yang, ShaoqiongGhosh, RajatNguyen, Minh DucYang, ShengyuanGiacobbe, MaurizioNguyen, Nam-TrungYang, ShikuanGiakos, GeorgeNguyen, T. HienYang, ShimingGiani, AnnaritaNguyen, Tam V.Yang, ShuhuiGiardina, PaolaNguyen, ThuyYang, TingGiatrakos, NikosNi, BingbingYang, Wei-hsiungGiavazzi, FabioNi, BingbingYang, WentaoGibert, XavierNi, QiangYang, WuqiangGiberti, HermesNichols, AndyYang, WuqiangGibson, AnnNico, GiovanniYang, XiaoxiGiesko, TomaszNicoloso, Rodrigo Da SilveiraYang, XinmaiGikas, VassilisNie, KaimingYang, YezhouGil, EduardoNie, LimingYang, YiminGil, EmilioNie, ZDYang, YingchenGil, PabloNielsen, Jimmy JessenYang, YongGillich, Gilbert-RainerNielsen, KristianYang, YongzengGini, GiuseppinaNielsen, ShawnYang, YuanGiordani, MarcoNiese, FrankYang, YunzeGiordano, DomenicoNigro, MarialisaYang, ZhiGiorgetti, AndreaNiitsu, KiichiYang, Zhi-XinGiorgetti, AndreaNijsten, M.W.N.Yangui, SamiGiovenzana, ValentinaNikolelis, DimitrosYanushkevich, SvetlanaGiraud, FrédéricNiku, SaeedYao, JasonGirousi, StellaNilsson, Hans-ErikYao, JiafengGiubileo, GianfrancoNilsson, TobiasYao, JunjieGiudice, NicholasNing, LipengYao, LinGiuntoli, JacopoNing, TigangYao, LinaGjevestad, Jon GlennNishio, YoshifumiYao, Xin-WeiGlaser, StevenNishitani, NozomuYao, ZhengGlass, John D.Nishiyabu, RyuheiYap, Wai KeanGlass, Samuel V.Nishiyama, MichikoYardley, JaneGlaum, JuliaNiu, XinruiYared, RamiGlenn, David (Michael)Nixon, MarkYavari, EhsanGlentis, George-OthonNoack, BenjaminYavuz, MetinGlisic, BrankoNocerino, EricaYe, FanGlösekötter, PeterNoda, KentarouYe, MaoGlover, IanNoh, GosanYe, ZhengmaoGlowacz, AdamNoh, YohanYedlin, MatthewGlynn, Macdara T.Nori, FrancescoYeh, Chien-HungGmyrek, ZbigniewNorouzi, HamidYeh, Kuo-HuiGnade, Bruce E.Norrdine, AbdelmoumenYeh, Lu-ChenGnyp, Martin L.NOSHADI, AminYeh, Sheng-ChengGockenbach, ErnstNossol, EdsonYeh, Wei-ChangGodah, WalyeldeenNounou, Mohamed IsmailYehezkeli, OmerGodfrey, AlanNour, OmerYehezkeli, OmerGodinez Tello, RichardNovak, DomenYen, Chih-TaGoethals, PauwelNovelli, AntonioYen, Jesse T.Goh, CindyNowak, AleksanderYen, Jia-YushGoh, Shu TingNowak, LechYeo, Woon-HongGoiffon, VincentNtalampiras, StavrosYeoh, PheeGolen, Erik F.Numata, DaijuYi, JaeseokGollmann, DieterNunez, A.Yilmazer, NuriGolonka, LeszekNuñez, NeftaliYim, SeongjinGoltz, Douglas M.Nuzzolese, Andrea GiovanniYim, YongbinGolub, EyalNychas, George-John E.Yin, WuliangGomes, DanieloNyka, KrzysztofYin, YouGómez, LuisNykiel, GrzegorzYin, ZhaozhengGómez, VicençO’Connell, BrianYing, BidiGomez-Barrero, MartaO’Mahony, ConorYing, GuodongGomez-Bravo, FernandoObare, SherineYoh, ShikohGomez-Clapers, JoanObeid, SimonYoneyama, SatoruGómez-de-Gabriel, Jesús M.Obien, Marie Engelene J.Yong, Yang-ChunGomis-Bresco, JordiO’Brien, AndrewYong, YuenkuanGonçalves, GilObst, MarcusYoo, ByungseokGonçalves, Luiz M. G.Occhipinti, LuigiYoo, Chang-SunGonçalves, Paulo Jorge SequeiraO’Connell, EoinYoo, HongseokGonçalves, RicardoOdeh, Suhail M.Yoo, SeehwanGonen, BilalOdijk, DennisYoo, Seong-eunGong, ChongfengOdolinski, RobertYoo, Wook JaeGong, JiaqiOdom, James VernonYoon, HargsoonGong, JieO’Driscoll, CillianYoon, Hyeun JoongGong, Nan-WeiO’Droma, MairtinYoon, JongheeGong, ShaofangOesterschulze, EgbertYoon, Kwang-seokGong, WeiOffermans, PeterYoon, SangpilGong, XiaowenOgiela, MarekYoon, SeokhoGong, YongkangOgurtsov, VladimirYoon, SeokhoonGonzalez, JoaquinOh, Beom-SeokYoon, SeongkyuGonzalez, Luis SanchezOh, HeekuckYoon, Yeo-SunGonzalez, MiguelOh, Hyun-UngYoon, Yong-IkGonzalez, Nelson MimuraOh, Min-cheolYoshimoto, ShigekaGonzalez, RubenOh, Sang-HyunYoshinobu, TatsuoGonzález, AlejandroO’Hare, DannyYou, TianyanGonzález, Antonio GuerreroOhhashi, AkiyoshiYounes, Maram BaniGonzalez De Los Reyes, Rafael CorsinoOho, ShigeruYounis, AdnanGonzález Dugo, VictoriaOhodnicki, Paul R.Yousaf, AdnanGonzalez Madruga, DanielOhya, HiroyoYousefi, SiavashGonzález Pascual, CésarOikonomou, GeorgeYperzeele, LaetitiaGonzalez Ruiz, VicenteOjeda, LauroYu, BinGonzalez-Valdes, BorjaOkada, HikaruYu, Chin-pingGoodarzi, Farhad A.Okarma, KrzysztofYu, ChunyangGoodin, Douglas G.Okarma, KrzysztofYu, FabiaoGoossen, KeithOkayasu, TakashiYu, FeiGopalakrishnan, BhaskaranO’Keefe, StevenYu, GangGöpfert, BeatOken, Barry S.Yu, GuanghuiGorazd, ŠtumbergerOku, YoshitakaYu, GuihuaGöröcs, Zoltán S.Okubo, MasaakiYu, GuizhenGorodetsky, AndreiOlaru, AndreiYu, Gwo-JongGorricho, Juan-LuisOldham, KennYu, Gwo-JongGorski, WaldemarOlivares-Mendez, Miguel AYu, HaoranGórski, MarcinOliveira, Elcio C.Yu, HongmeiGorte, BenOliveira, HenriqueYu, HongxiangGosalbez, JorgeOliveira, João P.Yu, HongyuGosselin, BernardOliveira, João F.Yu, HuapengGostar, Amirali KhodadadianOliveira, LuísYu, JiangboGoswami, KalyanOliveira, Tiago RouxYu, NenghaiGotsis, Konstantinos A.Oliveira Salles, MaiaraYu, Son-cheolGötzelmann, TimoOliver, NickYu, Ting-ToGoudos, SotiriosOller, JoaquimYu, WenxianGoursaud, ClaireOlmos, A. MartínezYu, XiangGouton, PierreOlsen, Lars FolkeYu, ZeyunGoverni, LapoOlsen, RasmusYu, ZhangweiGraaf, H Van DerOlthuis, W.Yuan, FeiGrace, PaulOlyaee, SaeedYuan, JunqiGracias, DavidO’Mahony, JosephYuan, LeiGradziel, Marcin L.Omar, NoshiYuan, QiangqiangGraeff, Carlos F. O.Omitaomu, Olufemi A.Yuan, QiangqiangGräff, ThomasOmosebi, AyokunleYuan, ShenfangGraham, GaryOmurtag, AhmetYuan, YinquanGraichen, UweOnen, OnursalYuan, ZhaoGrammalidis, NikosOneto, LucaYuce, MehmetGranado, Otoniel LopezOng, Eng-JonYue, ShigangGranda, Juan CarlosOniga, FlorinYun, YoungsunGranelli, FabrizioOno, ShintaroYurkiv, VitaliyGranger, MichaelOnori, MauroYusa, NoritakaGranitto, Pablo M.Opromolla, RobertoZabalza, JaimeGrant, JamesOrbach, RonZaccheroni, NelsiGrau, AntoniOrbaek White, AlvinZafra, AmeliaGravina, RaffaeleO’Reilly, ColinZagar, BernhardGravina, RaffaeleOren, YoramZagrodny, BartłomiejGravina, RaffaeleOrlandini, AndreaZaharis, Zaharias D.Grayson, MatthewOrlando, CalogeroZaidan, Martha ArbayaniGrebby, StephenOrlando, LucianaZaidi, Shabi AbbasGreen, David R.Orlinskii, SergeiZalama, EduardoGreen, NathanOrniana Ancuti, CodrutaZalyubovskiy, VyacheslavGreen, RylieOrr, Brian J.Zamora-Cristales, ReneGregory, Mark A.Ortega, Francisco R.Zanardi, ChiaraGrenzdörffer, GörresOrtiz, AlbertoZander, StefanGreve, DavidOsaba, EnekoZanetti, Sidney SáraGrill, RomanOsborne, FrancescoZanon, MarioGritzalis, ThanosO’Shaughnessy, SeamusZapién, Hiram GaleanaGrobnic, DanOsinska-Skotak, KatarzynaZappatore, MarcoGroen, Thomas A.Osman, Hussein AlZappatore, MarcoGross, Jason N.Ospina, GustavoZappino, EnricoGrøtli, Esten IngarOstasevicius, VytautasZarejousheghani, MashaalahGruber, JonasOstrowska, KseniaZarifi, Mohammad H.Grujić, Zoran DraganOsuch, TomaszZarpelão, BrunoGrulkowski, IreneuszOsypiuk, RafałZeng, GuangminGrünberger, AlexanderOszust, MariuszZeng, XinanGrzegorzek, MarcinOtaki, MasahiroZeng, YifengGu, ChangzhanOtis, MartinZeng, ZhoumoGu, FengshouOton, ClaudioZennaro, MarcoGu, FuqiangOtsuka, YuichiZese, RiccardoGu, LinjiaOu, Chia-HoZevenbergen, Marcel A. G.Gu, QijunOu, JianliangZhan, LingweiGu, Qun JaneOu, JianzhenZhan, NaiqianGu, YuOude Elberink, SanderZhang, Ai-DongGu, YujieOuyang, YuanxinZhang, Amy H.Guan, BinØvsthus, KnutZhang, AnruGuan, DonghaiO’young, SiuZhang, BaochengGuan, HaiyanOzdemir, SahinZhang, BiaoGuan, QuanshengÖzel, ÖmürZhang, BoshengGuccione, PietroÖzel, R. EmrahZhang, BowuGucunski, NenadOzeri, ShaulZhang, BowuGUEMES, JESUS ALFREDOPaal, StephanieZhang, ChrisGuerra, RuiPaavola, MarkoZhang, DanGuerreiro, Bruno J. N.Pachauri, VivekZhang, DaqiangGuerrier, StéphanePacheco, AndréZhang, DaqingGuerrieri, AntonioPacheco, João G.Zhang, FeihuGuerriero, FrancescaPackirisamy, MuthukumaranZhang, FengjiaoGuidash, MichaelPacyna, PiotrZhang, GuangyanGuidetti, RiccardoPadget, JulianZhang, GuogangGuidi, FrancescoPaek, JeongyeupZhang, HaihongGuijt, Rosanne M.Paeng, Dong-GukZhang, HaijunGuise, NicholasPaffenholz, Jens-AndréZhang, HaijunGuitton, AlexandrePAGANA, GuidoZhang, HaoGuivant, JosePaglialonga, AlessiaZhang, HeyeGuivant, JosePagliara, StefanoZhang, HongGuldiken, RasimPaiement, AdelineZhang, HongGuldiken, RasimPaipetis, Alkis S.Zhang, HouxiangGuldogan, Mehmet BurakPaiva, SaraZhang, HuGulinatti, AngeloPajovic, MilutinZhang, HuihuiGun’ko, YuriiPak, Jung MinZhang, JianrongGunawidjaja, RayPal, AnamitraZhang, JianyingGunther, JacobPal, SudeshnaZhang, JieGüntner, AndreasPalacin, JordiZhang, JingJingGuo, BinPalacios, AntonioZhang, JingtuoGuo, FeiPalafox, LeonZhang, JinjinGuo, QingboPalamartchouk, KirillZhang, JinjunGuo, QinghuaPaleari, MarcoZhang, JinshuiGuo, SongPalego, CristianoZhang, JueGuo, TuanPalella, Boris IgorZhang, JulieGuo, WeiPalleschi, VincenzoZhang, KuanGuo, WenzhongPalli, GianlucaZhang, KuanGuo, XiaonanPalmer, JamesZhang, LefeiGuo, XinxinPalmerini, Giovanni B.Zhang, LeiGuo, YanhuiPalmerini, LucaZhang, LeiGupta, Gourab SenPalmieri, LucaZhang, LiangpeiGupta, InderPalmiro, PoltronieriZhang, LihongGurkan, Umut AtakanPalomares-Salas, José CarlosZhang, NingGustafsson, PeikPalzer, StefanZhang, PengGutierrez, JairoPan, FeifeiZhang, QiGutiérrez, Juan ManuelPan, Jae-KyungZhang, QianniGutierrez-Blanco, OscarPan, LiyangZhang, QichunGutjahr, KarlheinzPan, YunZhang, QijinGuy, JonathanPanagiotakis, CostasZhang, QingGuzmán, PaulaPancheri, LucioZhang, RuiGyongy, IstvanPancheri, LucioZhang, ShanshanGyrard, AmeliePandarese, GiuseppeZhang, ShaolinKemp, Andrew H.Pandis, ChristosZhang, ShuleiHa, IlkyuPang, WeiZHANG, SongsongHa, MinkeunPang, XiaodanZhang, TaoHaas, RüdigerPang, ZhiboZhang, TingtingHabisreuther, TobiasPanneton, BernardZhang, TingtingHaddadi, KamelPanneton, BernardZhang, TongHadjiefthymiades, StathesPanousopoulou, AthanasiaZhang, WeipingHaesaert, GeertPanoutsos, GeorgeZhang, WeizheHahn, MichaelPantano, Maria F.Zhang, WenjiHaick, HossamPantazis, Dimos N.Zhang, WentaoHaick, HossamPaoli, AlessandroZhang, X.M.Haidar, AhmadPaolo, BellavistaZhang, XiaodongHaider, NomanPapa, Joao PauloZhang, XiaoshuanHaji-Sheikh, Michael J.Papadopoulos, Georgios Z.Zhang, XiaotongHakiri, AkramPapaelias, MayorkinosZhang, XiaozhuHamadache, MoussaPapageorgiou, Xanthi S.Zhang, XinfengHamadi, Ayoub AIPapaioannou, AthanasiosZhang, XinMingHamann, EliasPapkovsky, Dmitri B.Zhang, XinxingHamarneh, GhassanPappas, NikolaosZhang, XiruiHan, DongsooParadells, JosepZhang, XuHan, Gi-TaeParavati, GianlucaZhang, XuetaoHan, HyangsunParcharidis, IsaakZhang, YanpingHan, Il SongPardo-Iguzquizab, E.Zhang, YanxiaHan, Jae-hungPardossi, AlbertoZhang, YapingHan, Jee EunParedes, HugoZhang, YiHan, JungongParedes, JacoboZhang, YileiHan, MingParekh, SapunZhang, YinHan, ShuaiParente, ClaudioZhang, YongHan, TaoPareschi, RemoZhang, YongguangHan, TaoParigger, Christian GZhang, YongjunHan, YoukyungPariota, LuigiZhang, YongminHanaor, DorianParisi, AlfioZhang, YudongHancke, GerhardPark, ByungwoonZhang, YuMingHäne, ChristianPark, ChansikZhang, YunboHanke, KlausPark, Cheol-SooZhang, YuqiangHanke, UlrikPark, DongheeZhang, YutingHansard, MilesPark, EdwardZhang, ZhenjiangHänsch, RonnyPark, Hyun SoonZhang, ZhiguoHanson, C. WilliamPark, Je-KyunZhang, ZhiqiangHanson, RonaldPark, Jin GyuZhang, ZhonghuaHao, GuangboPark, Joong YullZhang, ZhongpingHao, KuangrongPark, Joon-ShikZhang, ZimingHao, PengPark, JoontaekZhang, ZonghuaHao, QiangPark, Jung-DongZhao, ChaoyingHao, QunPark, JungwonZhao, ChihangHao, XianjunPark, JunyongZhao, ChunHarjani, RameshPark, K. R.Zhao, DiHarle, RobertPark, Kyoung YoulZhao, DongHärmä, Harri J.Park, Kyung-AeZhao, GuangchaoHarris, Frederic J.Park, NamjeZhao, JuziHarvey, David M.Park, SehyunZhao, LifanHarvey, RichardPark, Soon-yongZhao, LiyaHasebe, YasushiPark, YongKeun (Paul)Zhao, MingshanHashemi, MortezaPark, Yong-LaeZhao, QingyuanHashimoto, HiroshiPark, Young JuneZhao, ShiyuHashimoto, Ken-yaPark, YoungilZhao, ShiyuHashimoto, MasanoriParker, RichardZhao, WeiHashimoto, MichinaoParmehr, Ebadat G.Zhao, WeishengHashizume, HiromichiParra, Andrés ConcaZhao, WenbingHassan, EmadeldeenParra, IgnacioZhao, XinHassan, ModarParrein, BenoîtZhao, YanjunHassanpour, SorooshParrilla, LuisZhao, YingweiHaubelt, ChristianParry, DaveZhao, YongHauglin, MariusPasciuto, IlariaZhao, YubinHaun, JeredPasero, ErosZhao, YulongHautefeuille, MathieuPasini, DarioZhao, ZhanHaux, ReinholdPasluosta, CristianZhao, ZimingHavlík, JanPassian, AliZheng, CuieHawes, MatthewPastorelli, StefanoZheng, GanHayashi, KenshiPastrone, ClaudioZheng, HuiruHayashi, YasuakiPata, PetrZheng, JinchuanHayat, AkhtarPatanè, FabrizioZheng, PengHe, BinPatel, SanjayZheng, ShiqiangHe, DavidPatel, SaurinZheng, ShjieHe, DebiaoPateraki, MariaZheng, YuanjinHe, HongshengPathan, Al Sakib KhanZheng, YufengHe, JiboPatias, PetrosZhong, LihengHe, LiangPatimisco, PietroZhou, ChangjianHe, LiFengPatra, Hirak K.Zhou, GongjianHe, LinaPatrick, ErinZhou, HuiyuHe, MeiPatzold, StefanZhou, LiangHe, ShiboPau, GiovanniZhou, LiangHe, ShiboPauer, HendrikjeZhou, Meng ChuHe, SuiningPaulasto-Kröckel, MerviZhou, Miao-leiHe, TaoPaulmurugan, RamasamyZhou, MuHe, Xue-FengPaulusse, JosZhou, QinHe, YaqianPavanello, FabioZhou, WenHe, ZhePavliček, PavelZhou, XiaHe, ZhihaiPawlowski, AndrzejZhou, Xiao DongHeard, GarryPaya, LuisZhou, XiaoluHeidari, HadiPayne, Christopher JZhou, XiranHeijne, ErikPazderski, DariuszZhou, YufengHeimhuber, ValentinPaziewski, JacekZhou, ZhiHein, JoachimPazzi, Richard W.Zhou, ZimuHeinse, RobertPecorella, TommasoZhu, BangheHeipke, ChristianPecori, RiccardoZhu, ChengzhouHeitzinger, ClemensPedersen, HenrikZhu, GeorgeHellbrück, HorstPedrocchi, NicolaZhu, GuoqiangHelmholz, PetraPedrosa, PauloZhu, HaoshenHemelrijck, Danny VanPeduto, DarioZhu, HonghuHen Hu, YuPeham, ChristianZhu, HuayangHenault, Jean-MariePeiris, Kamaranga H. S.Zhu, LingliHenesey, Lawrence E.Pektonen, JoukoZhu, LingliHeng, LiangPelegrí-Sebastia, J.ZHU, Ming-chaoHenriques, Fernando M.A.Pellenz, JohannesZhu, RongHeo, Yong-SeokPelletier, MathewZhu, XiaofengHer, Shiuh-ChuanPemen, A.J.M.Zhu, XiaoshanHeredia-Jimenez, JosePeng, Chien-fangZhu, XiaoyangHermans, RodolfoPeng, DiZhu, YingyingHernandez, NoeliaPeng, JueZhu, ZheHernández, LuisPeng, Sheng-YuZhu, ZheHernández, VicentePeng, XiaojunZhu, ZhenHernandez Perdomo, WilmarPeng, ZhengZhu, ZhiweiHernández Sosa, José DanielPennacchi, PaoloZhuang, JunHernández-Andrés, JavierPensyl, RussellZhuang, YanHerout, AdamPenzel, ThomasZhuge, XiaodongHerranz, José Miguel JiménezPeppas, Kostas P.Zi, BinHerrera, Pedro JavierPepper, MatthewZielinski, OliverHerrera-May, AgustinPercannella, GennaroZilic, ZeljkoHerrero, Jesus GarciaPerdoch, MichalZilly, FrederikHerrero, PauPereira, AntónioZingaretti, PrimoHerrero-Agustín, José-LuisPereira, ArvindZinno, IvanaHerry, Christophe L.Pereira, GuilhermeZiolkowski, RobertHerzog, KatjaPerenzoni, MatteoZiviani, ArturHerzog, OttheinPerera, KithsiriZizzo, GaetanoHessling, MartinPeres, António ManuelZlatanov, NikolaHianik, TiborPeres, EmanuelZondlo, Mark AHianik, TiborPerez, Alfredo J.Zór, KingaHide, MichihiroPerez Solano, Juan J.Zou, FrankHill, DanielPérez-Jiménez, RafaelZou, LongfangHill-Cawthorne, GrantPérez-Jiménez, RafaelZou, QinHiller, SusanPerez-Peña, FernandoZou, QiongjingHilsmann, AnnaPerez-Ramirez, DanielZou, XiaoboHinds, GarethPerez-Ruiz, ManuelZou, XudongHiremath, Shivayogi V.Perez-Uribe, AndresZou, YiHiromori, AkihitoPerez-Vidal, CarlosZou, ZhiqiangHirt, ChristianPerrone, GuidoZude, ManuelaHirtle, Stephen C.Persico, Adriano RosarioZuffada, CinziaHirtz, MichaelPersijn, StefanZuo, ChaoHiscox, AprilPeruzzini, MargheritaZuo, PengHlaváčová, ZuzanaPescaru, DanZych, MarcinHo, Chao-chingPescaru, DanZygielbaum, ArthurHo, Chin KeongPescosolido, Loreto


